# Understanding and mitigating hydrogen embrittlement of steels: a review of experimental, modelling and design progress from atomistic to continuum

**DOI:** 10.1007/s10853-017-1978-5

**Published:** 2018-02-06

**Authors:** O. Barrera, D. Bombac, Y. Chen, T. D. Daff, E. Galindo-Nava, P. Gong, D. Haley, R. Horton, I. Katzarov, J. R. Kermode, C. Liverani, M. Stopher, F. Sweeney

**Affiliations:** 1 Oxford Brookes University, Wheatley Campus, Wheatley, Oxford, OX33 1HX UK; 2 Department of Engineering Science, University of Oxford, Parks Road, Oxford, OX1 3PJ UK; 3 Department of Materials Science and Metallurgy, University of Cambridge, 27 Charles Babbage Road, Cambridge, CB3 0FS UK; 4 Department of Materials, University of Oxford, Parks Road, Oxford, OX1 3PH UK; 5 Engineering Laboratory, University of Cambridge, Trumpington Street, Cambridge, CB2 1PZ UK; 6 Department of Materials Science and Engineering, University of Sheffield, Mappin Street, Sheffield, S1 3JD UK; 7 Department of Physics, Imperial College London, Prince Consort Road, London, SW7 2BB UK; 8 Department of Physics, King’s College London, Strand, London, WC2R 2LS UK; 9 Warwick Centre for Predictive Modelling, School of Engineering, University of Warwick, Coventry, CV4 7AL UK

## Abstract

Hydrogen embrittlement is a complex phenomenon, involving several length- and timescales, that affects a large class of metals. It can significantly reduce the ductility and load-bearing capacity and cause cracking and catastrophic brittle failures at stresses below the yield stress of susceptible materials. Despite a large research effort in attempting to understand the mechanisms of failure and in developing potential mitigating solutions, hydrogen embrittlement mechanisms are still not completely understood. There are controversial opinions in the literature regarding the underlying mechanisms and related experimental evidence supporting each of these theories. The aim of this paper is to provide a detailed review up to the current state of the art on the effect of hydrogen on the degradation of metals, with a particular focus on steels. Here, we describe the effect of hydrogen in steels from the atomistic to the continuum scale by reporting theoretical evidence supported by quantum calculation and modern experimental characterisation methods, macroscopic effects that influence the mechanical properties of steels and established damaging mechanisms for the embrittlement of steels. Furthermore, we give an insight into current approaches and new mitigation strategies used to design new steels resistant to hydrogen embrittlement.

## Introduction

Hydrogen has remarkable properties and has for quite some time been promoted as a fuel for a zero carbon future. It is the lightest element, making it a cornerstone of quantum mechanics as a rare example of an exactly solvable two body problem. Hydrogen can dissolve in most metals and alloys [62], and its interactions with crystal lattice features are a reason for concern in iron, steel, nickel, titanium, vanadium, zirconium, silicon and other metals used in engineering [3]. Problems connected to hydrogen in materials are manifested in numerous ways, e.g. stress corrosion cracking, hydrogen-induced cracking, hydride cracking and other deleterious effects, often leading to catastrophic fracture. Since catastrophic failure is unacceptable in engineering, hydrogen’s detrimental effects need to be reduced as much as possible. Hydrogen-induced degradation and all phenomena associated with its deleterious effects are known as hydrogen embrittlement (HE), implying a hydrogen-induced transition from ductile to brittle behaviour. Research into the effects of hydrogen on mechanical properties was launched at the end of the nineteenth century by studying effects on iron and steels by Johnson [86] and later confirmed by Reynolds [174]. Since then, the effect of H-assisted degradation has been widely researched and explained [11, 35, 213, 241]. However, the underlying mechanisms are still being discussed with numerous theories suggested and extensively reviewed [30, 123, 138, 169, 178, 227]. Due to complexity of the HE phenomena, it is required to have knowledge of the hydrogen interactions on the metal surface, how it enters the metal, its transport through the crystal lattice, its interaction with crystal defects (vacancies, dislocations, grain boundaries, solutes) and precipitates, inclusions, interfaces, etc., and most importantly, its effect on the modification of material properties. Experimental studies on HE have shown that the presence of H atoms in a solid has a large influence on both crack nucleation and propagation [227]. It is established that HE is connected to the fast diffusion of H atoms through the solid materials lattice, often by quantum mechanical tunnelling even at room temperature followed by interaction with crystal defects. These interactions are often dominant in determining the influence of H on metals. Moreover, interactions of hydrogen with crystal defects and its influence are fundamentally less understood than diffusion of hydrogen in the perfect crystal lattice.

It is common to refer to H atoms in the lattice as diffusible hydrogen,^1^ which moves through the normal interstitial sites in the crystal lattice, and trapped hydrogen, which is non diffusible and resides around various crystal imperfections. These imperfections serve as traps for H atoms, which include vacancies, dislocations, grain boundaries, solutes, precipitates, inclusions, interfaces [138, 163, 165]. Hydrogen trapped around lattice defects can be roughly divided into two subcategories: (a) strongly trapped hydrogen, residing in irreversible or deep traps (solutes, precipitates, inclusions, where hydrogen has a low probability of escape due to the large potential energy barrier that must be overcome to do so) which will not be released during service, and (b) weakly trapped hydrogen residing in reversible or shallow traps which can be released during service (i.e. the probability of escape is higher, associated with a lower potential energy barrier). When H is released from reversible traps it can cause further damage in already degraded material.

The initiative for this paper came from researchers working in the HEmS (Hydrogen in metals from fundamentals to the design of new steels) programme grant, funded by the Engineering and Physical Sciences Research Council. The focus of this major initiative on hydrogen embrittlement is to develop a deep understanding of the effect of hydrogen in metals by covering all of the aspects from atomistic simulations to continuum modelling and by adopting recently developed advanced characterisation and testing procedures. One of the most important aims of the HEmS programme grant is the design of a new family of high-strength steels resistant to hydrogen embrittlement.

The objective of this paper is to give a reader insight into the changes caused by hydrogen residing in materials, a process that can have catastrophic effects. We mainly focus on iron and steels; other metal systems are briefly introduced to explain additional effects that the presence of hydrogen can cause. Following catastrophic fractures related to hydrogen we will then discuss where hydrogen is likely to reside and how hydrogen moves inside the material. The detection of hydrogen inside the material is extremely challenging. We believe that combining experimental, numerical and theoretical techniques is the way forward to make a breakthrough in understanding hydrogen embrittlement in metals. We discuss relevant experimental methods available in the literature, such as thermal desorption analysis (TDA) and permeation experiments used to monitor ingress and egress of hydrogen. We then discuss innovative experimental techniques used to detect hydrogen inside the material, such as atom probe tomography (APT) or high-resolution electron microscopy. Atomistic calculations are then introduced as they provide insight into stable positions where hydrogen resides and microscopic mechanisms of hydrogen diffusion. "Description of the failure mechanisms affecting steels" section is devoted to a review of accepted hydrogen embrittlement mechanisms using recent experimental evidence and modelling techniques at different length scales. It is widely accepted that in most cases a combination of mechanisms, activated under different conditions, is responsible for HE. The key point is to understand under which conditions these mechanisms are activated. Despite the amount of research dedicated to this topic, this point remains elusive. This is an essential point from a steel design point of view, i.e. how can we design HE resistant steels if we do not have a clear picture of what are the mechanisms of failure caused by hydrogen in a given steel? We dedicate "Hydrogen embrittlement mitigation strategies and design of new steels" section to hydrogen mitigation strategies. We give a detailed overview on strategies adopted in order to mitigate HE. We emphasise the use of engineered microstructures, which contain hydrogen traps, with the scope of reducing the concentration of diffusible hydrogen (e.g. [164, 166]) in steels.

## Hydrogen in steels

In this section we first discuss experimental techniques and kinetic models to measure ingress and egress of hydrogen. We then dedicate a section to innovative experimental techniques used to detect hydrogen inside the material, such as atom probe tomography (APT) or high-resolution electron microscopy. Atomistic calculations are then introduced as they provide invaluable insight into stable positions where hydrogen resides and microscopic mechanisms of hydrogen diffusion inside the material.

### Kinetics of hydrogen in iron and steels

We dedicate the subsection below to the description of how diffusible hydrogen atoms move inside the material. In particular we discuss up-to-date experimental techniques and kinetic models used to interpret measurements of ingress and egress of hydrogen in metals.

#### Experimental techniques and hydrogen kinetic models to measure ingress and egress of hydrogen in metals

Absorption of hydrogen in pure iron exceeds the solubility limit due to trapping at defects such as vacancies, dislocations, grain boundaries, inclusions, precipitates and other features. In order to evaluate the quantity of hydrogen within a metal, and the effects of trapping sites, several methods have been developed. The most commonly used is method thermal desorption analysis (TDA) where the hydrogen desorption rate is measured as a function of temperature. The binding energies of traps and their corresponding specific hydrogen capacities are quantified from measured results. Samples are first charged, either electrochemically or in a high-pressure chamber with ${\hbox {H}}_2$ gas, with the aim of saturating the traps. The sample is then put into a tube furnace connected to a gas chromatograph to detect the amount of released hydrogen. In order to assure that hydrogen is completely degassed, the sample is heated at a constant rate. TDA is also used in order to characterise the amount of H present in a component in both (a) "as-delivered" condition or (b) after a certain time in service. Several methods can be used to analyse the results. Here they are summarised into three categories based on the underlying properties: (a) reaction kinetics model [29, 106], (b) McNabb–Foster trapping–detrapping model [128] and (c) hydrogen local equilibrium model [153].

- The reaction kinetics model for hydrogen trapping was proposed by Choo et al. [29] where the detrapping activation energy is determined from the peak temperature $T_{\mathrm{p}}$. The desorption kinetics is given by the following equation:
  1 $$\begin{aligned} \frac{\partial X}{\partial t}=A\left( 1-X\right) \exp \left( \frac{-E_{\mathrm{a}}}{RT} \right) \end{aligned}$$
  In this equation, *X* is fraction of hydrogen released [equal to $(\frac{H_0 - H_t}{H_0})$] and is calculated as a function of the proportionality constant *A*, gas constant *R* and temperature *T* at time *t*. $E_{\mathrm{a}}$ is the activation energy needed to escape from a trapping site, $H_0$ is the amount of H in trapping sites at $t=0$ and $H_t$ is the amount of H at time $t\ne 0$. When using a constant heating rate $\phi$, the maximum desorption rate is obtained when the derivative of Eq. (1) is zero. The trap energy is then calculated as:
  2 $$\begin{aligned} \frac{\partial \left( \phi /{T_{\mathrm{p}}^2} \right) }{\partial \left( {1}/{T_{\mathrm{p}}} \right) }=\frac{-E_{\mathrm{a}}}{R} \end{aligned}$$
  The reaction kinetics model has been extensively used to analyse TDA results [29, 112, 206, 230]; however, the comparison of the results from various sources is not straightforward. Furthermore, the model is based upon the kinetics of homogeneous chemical reactions, and it does not take hydrogen diffusion into account. Moreover, there is no sample size-dependence or trap density in the model above. Therefore, this model is not applicable in samples for which diffusion is the limiting factor instead of the liberation of hydrogen from the traps. Recently this model has been improved by various researchers to include more than one type of trap and size dependency of samples [34, 101, 195].
- Another hydrogen diffusion model was proposed by McNabb and Foster [128] which includes the kinetics of trapping and detrapping, the activation energy law and mass conservation:
  3 $$\begin{aligned} \frac{{\mathrm{d}} \theta _{\mathrm{t}}}{{\mathrm{d}}t}= k \theta _{\mathrm{l}} (1-\theta _{\mathrm{t}}) - p \theta _{\mathrm{t}} \end{aligned}$$
  where the hydrogen occupancy, $\theta$, is defined as $\theta _i=C_i/N_{\mathrm{t}}$, i.e. the concentration of traps, $C_{\mathrm{t}}$, or lattice sites, $C_{\mathrm{l}}$, divided by the trap density, $N_{\mathrm{t}}$, and where *k* and *p* are factors representing the rate at which H atoms are either trapped or escape from a trap as per:
  4 $$\begin{aligned} k=k_0 \exp \left( \frac{-E_{\mathrm{D}}}{RT} \right) p=p_0 \exp \left( \frac{-\,(E_{\mathrm{D}}+E_{\mathrm{b}})}{RT} \right) \end{aligned}$$
  where $k_0$ and $p_0$ are pre-exponential factors, $E_{\mathrm{D}}$ is the activation energy for diffusion and $E_{\mathrm{b}}$ is the trap binding energy. Due to the large number of fitting parameters ($k_0, p_0, N_{\mathrm{T}}, E_{\mathrm{b}}$), the determination of binding energy is extremely difficult. The model in Eqs. 3 and 4 has also been utilised to analyse TDA results [43, 44, 47, 210, 216].
- Oriani [153] proposed a model where a local equilibrium of hydrogen in the traps and lattice is assumed. Diffusion in the lattice is given by the following equations:
  5 $$\begin{aligned} \frac{{\mathrm{d}} C_\mathrm{{l}}}{{\mathrm{d}} t} + \frac{{\mathrm{d}} C_\mathrm{{t}}}{{\mathrm{d}} t} = D_\mathrm{{L}} \frac{{\mathrm{d C}}_{l}^{2}}{{\mathrm{d}}x^2} \end{aligned}$$
  where $D_{\mathrm{L}}$ is the lattice diffusivity. Assuming $\theta _{\mathrm{l}} \ll 1$, occupied lattice and trapped sites are in equilibrium when
  6 $$\begin{aligned} \frac{\theta _{\mathrm{t}}}{1-\theta _{\mathrm{t}}}=\frac{\theta _{\mathrm{l}}}{1-\theta _{\mathrm{l}}} \exp \left( \frac{-E_{\mathrm{a}}}{k T}\right) \end{aligned}$$
  The assumption $\theta _l \ll 1$ is needed and is reasonable, since the hydrogen concentration in the lattice is generally very low. Bombac et al. [21] verified the assumption of local equilibrium of hydrogen in the traps and lattice by using quantum mechanically informed kinetic Monte Carlo simulations in defective microstructures.

Another technique used to experimentally determine the trap behaviour is the measurement of permeability rate through a metallic membrane. In order to measure the hydrogen permeation rate in metals, a Devanathan–Stachurski (DS) [38] cell is widely used. The DS Cell is a system with two individual electrolytic cells, separated by the investigated sample (membrane). Hydrogen is produced electrochemically in the cathodic compartment and absorbed at the sample surface before diffusing through the bulk of the sample. In the anodic compartment (exit side) hydrogen that has diffused through the sample is oxidised at a constant potential. The anodic current is measured throughout the experiment. The amount of hydrogen that diffuses through the sample is proportional to the measured oxidation current. Since currents can be accurately measured, the DS method is a very sensitive and accurate method of determining the hydrogen flux as a function of time. Devanathan and Stachurski [38] based their technique on the assumptions that (a) the conditions at each of the compartment membrane surfaces are well defined and are established under potentiostatic control and (b) impurities and surface effects (including recombination of H atoms to ${\mathrm{H}}_{2}$) are negligible compared to the bulk diffusion:

- Coverage of the sample surface on the absorption side is constant and zero on the exit side.
- Hydrogen adsorption kinetics is fast enough to maintain equilibrium at the absorption side of the sample.
- The hydrogen permeating through the sample is immediately oxidised at the exit surface.

In order to fulfil all of the assumptions stated, experiments need to be performed very carefully and are difficult to compare across different studies. During experiments the time needed to obtain hydrogen on the exit side of the sample surface is termed the breakthrough time $t_{\mathrm{b}}$. After breakthrough, the permeation current increases until a steady state condition is reached, where flux of hydrogen is in equilibrium ($J_{\mathrm{SS}}$) and the current reaches a plateau. This allows determination of the surface hydrogen concentration [38].

### Preferred sites for hydrogen

Direct atomic-scale evidence for hydrogen is limited due to the fact that many of the standard imaging techniques are not suitable for the analysis of hydrogen. We dedicate the subsection below to describing recent advances in experimental techniques used to detect hydrogen atoms in metals.

#### Experimental techniques to detect hydrogen in metals

Two common techniques, x-ray and electron diffraction, are arguably the standard materials analysis methods, but they do not interact appreciably with hydrogen. This lack of interaction is due to the combination of the hydrogen’s low electron and x-ray cross section, which originates due to the small electron cloud [129]. This effectively makes hydrogen largely transparent to electron and x-ray radiation. Moreover, hydrogen exhibits a high diffusivity. Hence, hydrogen is difficult to analyse since at sub-micron scales it can be mobilised in many materials under natural experimental conditions.

In steels, in particular, this is further complicated by the low solubility of hydrogen, which under normal conditions may be on the order of ppm. This places even greater constraints on methods such as energy dispersive spectroscopy or energy loss spectroscopy, which typically require $>0.1$ at.% for detection [148]. To the authors’ knowledge there is currently no single best technique for imaging hydrogen within a material. A combination of indirect approaches is used to understand the interaction of hydrogen with a target material at the nanoscale. In order to perform effective imaging, one must look to alternative and usually more costly analysis techniques.

One possible approach is the use of neutron scattering. Unlike x-ray or electron methods, neutron scattering cross sections for the most dominant isotope, ${}^{1}\hbox {H}$ (hydrogen-1), are appreciable with an incoherent cross section of $80.27 \times 10^{-24} {\mathrm {cm}}^2$—as a comparison, isotopically weighted iron has a cross section of only $11.62 \times 10^{-24} {\mathrm {cm}}^2$ [136]. This implies that (to a first approximation), a hydrogen nucleus will scatter 8 times more strongly than iron in a steel. As such, this can be a powerful tool for hydrogen analysis, such as in protein crystallography and hydride analysis. Neutron techniques have also been employed in examining steels [125, 219], where one should expect that with the addition of hydrogen, the total scattering should increase. Indeed, this is observed in small-angle neutron scattering (SANS) data, where charged samples of finely dispersed vanadium carbide precipitates appear to slightly increase the total scattered intensity after hydrogen-1 charging [152], and after subsequent annealing, the scattered intensity is reduced back to the uncharged state. Ohnuma [152] suggests that their approach could detect hydrogen at 0.03 at.% levels. It is noted that the signal levels involved in these works are not very high, compared to the scatter in the data (although they are definitely resolved), indicating the difficulty of imaging such low concentrations in these materials.

The disadvantages of the method are, however, quite clear. This technique requires access to expensive neutron sources, which are not typically available to modest laboratories at a high-throughput scale. Secondly, this is a diffraction technique with a difficult to control beam, due to its naturally low interaction as compared to x-rays or electron sources. The mean free path of a thermal neutron in a strong absorber, such as ${}^{6}{\mathrm {Li}}_{2}{}^{10}{\mathrm {B}}_{6}$, is of the order of $40 \, \upmu \hbox {m}$ [27], where for an electron in a strong absorber, such as gold, this is approximately 9 nm [88]. This vast difference implies that imaging individual interactions of hydrogen to given microstructural features with neutron imaging is non-trivial, as only very low intensities will be observed. Rather, aggregations of interactions must be observed to obtain a meaningful signal.

**Figure 1:**
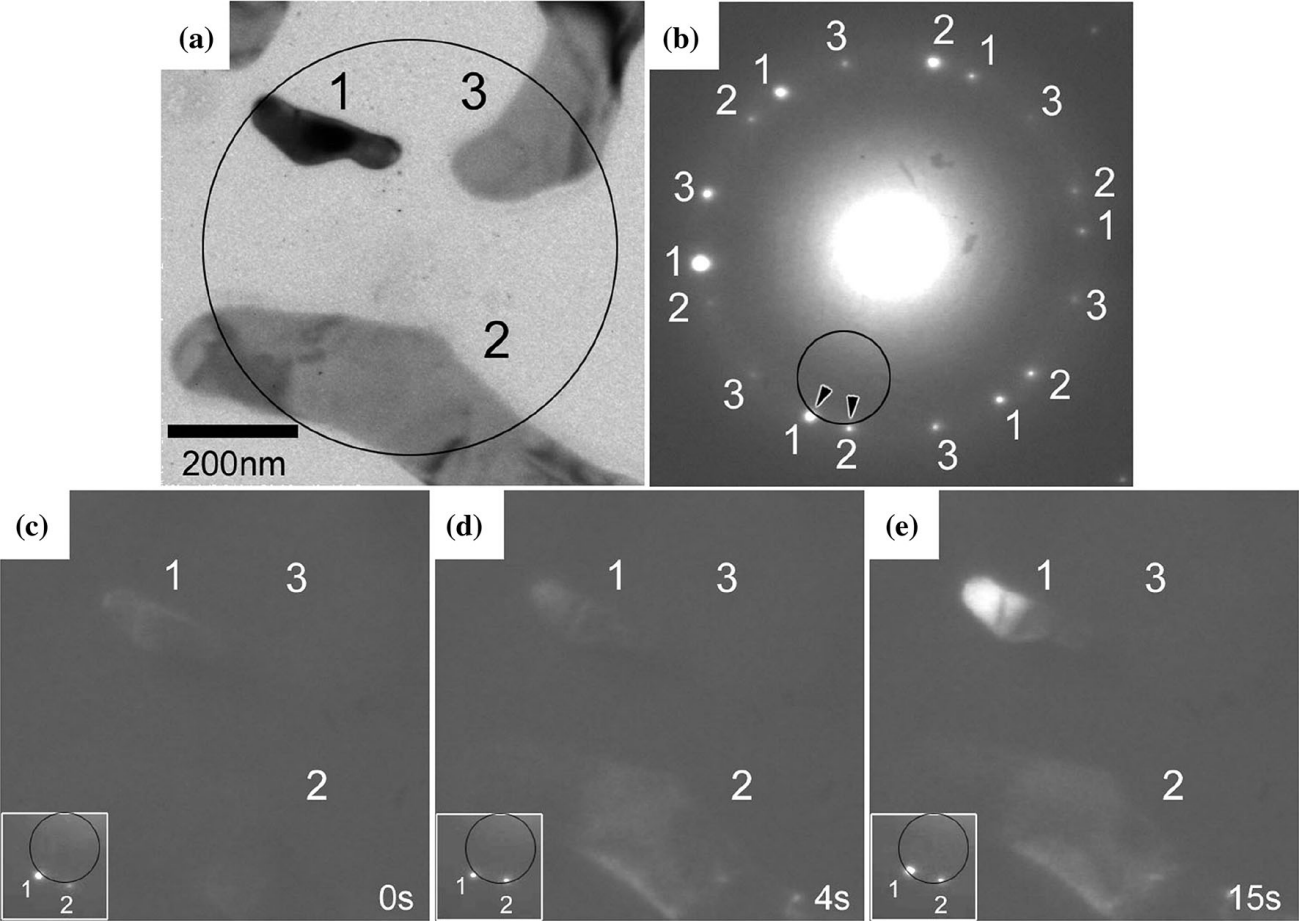
**a** and **b** Bright Field image and SAED pattern at 0.8 bar, respectively. The SAED pattern was taken from the area in the black circle in **a**. **c**–**e** Dark-field images taken 0, 4 and 15 s after decreasing the temperature from 140 °C to room temperature. Subfigure **e** shows diffraction contrast from SAED using additional spots from hydrogen-charged foil

Imaging of hydrogen in transmission electron microscopy (TEM) has been performed, but only in very specific situations, such as on graphene films [129]; hence, the technique does not expand to generalised imaging. TEM imaging of hydrogen atoms in other specific situations has also been claimed, such as for hydrides, both at the macroscopic [168] and at the atomic scale [81]. High concentrations of hydrogen do provide contrast indirectly, as shown in Fig. 1. This can either be due to an alteration of the phase comparative to the surrounding material [235], or via changes in the plasmon peak [73].

Clearly, TEM is not the most effective method for the detection of low concentrations of hydrogen within a bulk material, or hydrogen segregated to complex features, such as dislocations. Nevertheless, experimental evidence for hydrogen interaction with dislocations in TEM has been achieved by careful exposure of TEM foils to hydrogen within a specialised environmental cell [178], which initiates otherwise stationary dislocations to undergo motion during exposure, and the dislocations cease moving upon removal of the environmental hydrogen. This effect was only reported for hydrogen, or gaseous mixtures with water vapour. This directly shows that, even at low hydrogen concentrations, the energy barriers to dislocation motion can be reduced. This result will be discussed in more detail in "Description of the failure mechanisms affecting steels" section.

An alternative method for imaging at microscopic ($\upmu \hbox {m}$) length scales is an indirect technique known as microprinting. In this technique hydrogen is diffused through the material, and made to interact with a chemical species, such as silver, in order to form silver crystals in regions where hydrogen is present [155]. Such images often show silver formation at grain boundaries or surrounding inclusions, leading to the concept of preferential diffusion at or near such microstructural features. It is thus surmised that this may be a pathway through which hydrogen can enter a material at higher rates than suggested by bulk diffusion analyses. Similarly, Pd films have been utilised to form hydrides, which can be examined by scanning probe methods [48].

These methods are an extension of the Kelvin probe methods, which utilise a an atomic force microscopy (AFM) needle, in conjunction with an applied oscillating electric potential, in order to measure the potential between the probe and the sample surface [49]. In conjunction with control of the current, it is possible to measure the work function of the surface, $\Delta \Psi$, to high resolution [179]. The related scanning Kelvin probe force microscopy (SKPFM) allows for the extraction of information relevant to the hydrogen distribution in the form of this potential [189]. Experimentally, the measurement of hydrogen is achieved by diffusing hydrogen through the back of a sample (e.g. by electrochemical charging), and then measuring this work function at the front face, usually on a Pd-coated surface. Localised hydrogen enhancements appear as a temporally changing decrease in the SKPFM potential as hydrogen diffuses through the sample. Thus, not only do these measurements provide spatial, but also temporal resolution. Spatial resolutions on the order of 500 nm have been demonstrated [49].

There are several limitations to the method: firstly it is a surface technique and thus naturally does not provide 3D information. There are limitations on the accessible timescales, as with many other techniques, due to the time required to arrange the correct experimental configuration. There are some considerations required as to the charging intensity, as the Pd foil itself can saturate; however, this can be reasonably predicted. Lastly, the observed hydrogen distribution is a convolution of the hydrogen at the back plane of the sample and the propagation function through the sample. The technique, however, provides information which may not be present elsewhere, particularly with respect to the temporal data. This has been used to good effect in a duplex steel, showing the vastly different timescales of hydrogen propagation through ferritic and austenitic phases [189].

If imaging itself is ignored, and only depth information is required, bombardment reaction methods, such as energy recoil detection, can be utilised [110]. These methods involve bombarding a sample with a probe beam at MeV energy ranges, in order to create a nuclear reaction with specific target species within the host matrix. Common reactions include the ${}^{3}{\mathrm{He}}(d,p) {}^{4}{\mathrm{He}}$ reaction (7), as well as the (resonance) reaction (8).

7 $$\begin{aligned} {}^3 {\mathrm {He}} + {\mathrm {D}} \rightarrow {}^4{\mathrm {He}} + {\mathrm{proton}} + 18\,{\mathrm {MeV}} \end{aligned}$$

8 $$\begin{aligned} {}^{15} {\mathrm {N}} + {}^1 {\mathrm {H}} \rightarrow {}^{12} {\mathrm {C}} + {}^4 {\mathrm {He}} + \gamma _{\mathrm{ray}} \end{aligned}$$

In resonance reactions, the reaction primarily occurs when the ions are at a very specific energy. As the ions are travelling through the sample such that depth profile information arises from the loss in beam energy with sample depth. This implies that by scanning the beam, different depths can be accessed, and the total reaction cross section can be measured to yield concentration information. A detailed review of the technique is given by Wilde and Fukutani [232], where the primary limitations of the method are the achievable resolution within the crystal, and the sensitivity of the method.

To the authors’ knowledge, there is little direct observation of hydrogen at features such as dislocations and grain boundaries, which makes understanding the role of hydrogen more complex. One of the few techniques that claims to have an imaging capability is atom probe tomography (APT), albeit an indirect one. Atom probe functions by using immense electric fields, derived from high-curvature needle specimens, to cause evaporation of atoms. These are directed at a time-of-flight detector which can identify the mass-to-charge of the target species, regardless of the atomic type. The mass-to-charge can be related, usually unambiguously, back to the atomic species that was evaporated.

Whilst at first glance this seems optimal for hydrogen imaging, there is strong evidence that the hydrogen distribution observed by atom probe does not, in the general sense, relate to the true hydrogen distribution in the material. This is due to hydrogen from the vacuum system absorbing and re-emitting from the specimen surface, causing hydrogen to appear in the analysis, despite not being part of the specimen. Sundell has performed a detailed analysis of residual hydrogen, and its interaction with various APT experimental parameters [199]. Here it is demonstrated that the false H signal can easily be as high as 1.5 at.% in a NiCr alloy and is highly dependant on experimental conditions. It is essential that the H signal is not misinterpreted as microstructural data, even within a single dataset.

The primary technique for avoiding this problem is to utilise deuterium (${}^{2} \hbox {H}$) as an isotopic marker species. As deuterium has only a very low natural abundance (0.0115 at.%), it can be used as a tracer species. Deuterium detected within an atom probe experiment can be nearly unambiguously identified as part of the material, separating the vacuum system and specimen signal. This, however, is usually limited to voltage-mode experiments, as the laser causes complex molecular species, such as ionised molecules of $({}^1{\mathrm{H}})_{2}$ to form.

Deuterium studies have been undertaken by several authors [59, 64, 90, 156, 204, 208], often involving modifications to standard APT systems for introducing hydrogen into samples. These studies have shown that it is possible to image hydrogen within a range of materials; however, the APT is typically limited to only showing localised concentrations on the order of 20 at. ppm or higher. Primary concerns with this technique are related to the very small length scale (on the order of $100~{\mathrm{nm}}$) of the tips. Diffusion of hydrogen is thought to cause a severe reduction in signal for non-stable hydrogen compounds. Even in the case of deep traps, such as the TiC precipitates shown by Takahashi [203], cryogenic techniques were applied to minimise the diffusion loss.

Atom probe studies have been undertaken by several researchers, whereby, due to limitations in the detection of hydrogen-1 due to vacuum contamination, deuterium is used to load samples with hydrogen prior to analysis. Loading of hydrogen has been undertaken using gas-phase reactions, plasma methods and electrochemical loading. To date, extensive studies of the envelope within which experimental data can be successfully obtained are not yet, to the authors’ knowledge, available.

#### Quantum calculations to understand hydrogen sites and diffusion

Despite continuous progress on experimental techniques to detect hydrogen sites and to measure hydrogen kinetics, they are not alone sufficient to provide a complete insight on the mechanisms of HE. We believe that a combination of these high-resolution experimental tools and quantum calculations could give rise to a breakthrough in this field.

##### Theoretical evidences of hydrogen kinetics and hydrogen preferred sites

Atomistic simulation techniques allow one to question the favourability of a given arrangement of atoms within a given system. Thus, these techniques are perfectly placed to assess where hydrogen is likely to reside. It is worth noting that since iron is magnetic, and hydrogen is for all intents and purposes a quantum particle, great care must be taken in carrying out such simulations [40]. It is also worth noting that, due to the high computational cost of carrying out these quantum-mechanical simulations, there is a limitation on the size of the system that can be analysed. Furthermore, the choice of the simulations to be carried out often requires insight from experiments. This is due to the fact that the combinatorial scaling of the total number of possible simulations is extremely high. When assessing the effect of hydrogen in steels there are several iron phases of interest; the body-centred cubic phase (alpha iron or ferrite), the body-centred tetragonal phase (martensite), the face-centred cubic phase (austenite) and HCP martensite. Within the phases of interest of bulk iron, there are two high-symmetry sites; the octahedral and the tetrahedral. A number of authors have used density functional theory (DFT) to assess which of these sites is preferable for a given phase. This is achieved straightforwardly through calculation of the total energy of the system of iron with the hydrogen atom at the site of interest (the zero of energy can be made to be equivalent in both cases). These DFT simulations have shown that the hydrogen prefers the tetrahedral site over the octahedral site in both ferrite and martensite [31, 74, 85]. This is confirmed in the path-integral simulations by Gillan [61], which treat the hydrogen as a quantum particle, and show the bulk of the hydrogen probability distribution to be centred on the tetrahedral site for ferrite. Conversely in austenite the hydrogen prefers the octahedral site over the tetrahedral site [74]. These results are in agreement with experiments.

DFT calculations have been used in order to explain the phenomenon of hydrogen embrittlement and also the role of trapping sites for hydrogen as a possible solution to mitigate the phenomenon of hydrogen embrittlement in steels [31, 58, 70, 145, 146]. Detailed studies of H trapping at vacancies in both ferrite and austenite have been carried out. In both the BCC and FCC phase, vacancies are shown to strongly trap H; however, these traps are shown to be relatively short ranged as the interstitial sites next to the vacancy have a solvation energy that is almost indistinguishable from the bulk [145, 146]. Interestingly in BCC Fe the presence of a vacancy has been argued to change the preferred site for H from the T-site to the O-site [31]. In FCC, Fe vacancies have been shown to trap up to 6 H atoms and this phenomenon has been argued to be the driver behind the formation of super abundant vacancies [145, 146].

Due to the system size constraints on quantum mechanical calculations, there is limited work on grain boundaries and interfaces, with simulations often restricted to coherent/simple interfaces. Simulations carried out on $\Sigma 3$-type tilt grain boundaries of BCC and FCC show that in the BCC interface, interstitial sites have a more favourable solution energy than the lattice interstitial sites, and in the FCC grain boundary has a more endothermic energy than lattice interstitials. This suggests that the BCC boundary will attract H from the matrix whilst the FCC boundary will repel it. In the case of open grain boundaries, there is more variation in the interstitials at the boundary and as such a $\Sigma 5$ BCC and a $\Sigma 11$ FCC grain boundary are found to attract H [41, 74].

It has been observed that the addition of carbide precipitates (notably Ti and Nb) may improve resistance of steels to hydrogen embrittlement; however, investigations of the solution enthalpy of H in equiatomic bulk carbides (CrC, TiC, MoC, NbC, VC) have shown the enthalpy to be less than bulk Fe, meaning that H is unlikely to move into the bulk [74]. Investigations of ${\hbox {V}}_{4} {\hbox {C}}_{3}$ show promise as the carbon vacancies present in the bulk structure provide a deep trap for hydrogen [96]. It is worth noting that without calculating the energy barriers between the bulk Fe and the carbide interface and bulk it is hard to know whether hydrogen is likely to ever be in the vicinity of the trap site. Initial research indicates that many factors are involved in the trapping energies and barriers [39] Current steel designs containing carbide precipitates as traps for hydrogen are discussed in the last section of the paper.

##### Calculations of the hydrogen kinetics

It is well known that hydrogen is a species with one of the highest measured atomic diffusivities [225]; the notion that this might be enhanced by quantum tunnelling effects displayed by hydrogen, even at relatively high temperatures, has been known for a long time [231]. Nevertheless, entirely classical simulations have been employed to study the diffusion of hydrogen in iron, generally yielding results that appear in agreement with the experiments as long as the temperature is well above room temperature where quantum effects are less important (for example, see [61]).

The foundation of some of the best-known methods for performing approximate quantum dynamics calculations at present is the path-integral formulation of quantum mechanics (PIF) derived by Feynman [53]. This theory maps the quantum mechanical trace operation over the states of a particle onto a classical trace operation, in which the single particle is replaced by a closed loop of particles connected by harmonic, temperature-dependent springs. Whilst this quantum-classical isomorphism holds for equilibrium statistical mechanics, recently there have been significant efforts aimed at generalising this result to kinetics as well.

For the direct computation of dynamical quantities such as the trajectory of hydrogen within the iron lattice, the so-called centroid molecular dynamics (CMD) method is one such generalisation of the PIF. Originally devised by Cao and Voth [25], the CMD approach has proved useful for computing the diffusivity via the mean-squared-displacement formula. This methodology was employed, for example, to show how the dependance on temperature of the diffusion coefficient of hydrogen in BCC iron deviates from the classically predicted Arrhenius curve below about 500 K. When tunnelling effects effectively lower the transition energy barrier, the diffusivity increases [100]. The CMD method relies on the postulate that the dynamics of the *centroid* of the quantum particle, which is a quantity arising naturally in the PIF at equilibrium, carries information on the dynamics of the quantum particle itself. Efforts to further qualify this heuristic approximation from a theoretical perspective are still ongoing.

Another computational technique based on Feynman’s PIF was recently proposed by Craig and Manolopoulos [32]. Analogous to CMD, the ring-polymer molecular dynamics (RPMD) method exploits the correspondence between the quantum particle and the loop of classical particles; unlike CMD, RPMD samples the configurations in phase space by evolving the ring of classical particles thorough the Hamiltonian that can be read off the isomorphic classical trace [214]. Whilst this is again a heuristic method, RPMD has found a wide range of applications due to its conceptual and computational simplicity. It has been employed in the investigation of the diffusion process of hydrogen in BCC iron in a similar fashion to CMD. Results appear to confirm the importance of quantum effects at about 500 K and below [236].

Both the CMD and RPMD techniques rely on having a potential energy surface, which for the case of hydrogen in iron can be obtained by means of DFT, tight-binding calculations, or an interatomic potential method such as the embedded atom model.

An important application of RPMD is in the context of quantum-mechanical transition rate theory and transition-state theory; here the rate constant can be employed to obtain the diffusivity via Einstein’s relation. In particular RPMD can be employed to compute the ratio of partition functions involved in the transition-state expression of the rate constant, but also as a tool for evaluating directly the dynamical factor that needs to be computed in order to obtain the full transition rate from the transition-state expression. Efforts to justify the former approach rigorously have recently proved successful [72], and its use in the context of hydrogen diffusion in iron yields results in good agreement with experiments [94]. An interesting fact involving quantum transition rate theory was found by Katzarov et al. [92]: their recent work suggests that the transition-state theory approximation of the rate constant would be less reliable when quantum-mechanical effects become important. This is due to the fact that the dynamical correction encoded in the so-called *transmission coefficient* also takes into account tunnelling effects; these are once again found to be relevant at room temperature for hydrogen migrating in iron.

Overall, the PIF-based techniques employed for studying the kinetics of hydrogen in metals have been extremely useful for investigating the role played by quantum-mechanical effects in the diffusive process, and more theoretical and computational work should be carried out so as to exploit this powerful semiclassical framework even further.

### Discussion

In this section we have discussed a range of experimental, modelling and computational techniques currently used to detect hydrogen preferred sites and kinetic processes at different length scales. A combination of nanometre-scale experimental tools (e.g. TEM, Nano-SIMS and APT) and quantum calculations is essential to provide insight in elucidating the macroscopic effects that hydrogen has on the mechanics of steels. The next section is dedicated to an in-depth description of the HE mechanisms including supporting experimental and modelling evidence across different length scales.

## Description of the failure mechanisms affecting steels

There has been a substantial research effort dedicated to understanding the failure mechanisms in steels due to the presence of hydrogen. There are a number of hydrogen embrittlement mechanisms that have been proposed in the literature; however, there are still conflicting opinions among researchers. Despite there being a vast body of literature on HE mechanisms, a synthetic but detailed review of these mechanisms which also includes supporting experimental and modelling evidence across different length scales is still lacking in the authors’ opinion. Hereafter we focus on the following mechanisms: hydrogen-induced decohesion (HID), hydrogen-enhanced local plasticity (HELP), hydrogen-induced phase transformation (HIPT) and hydrogen-enhanced strain-induced vacancy formation (HESIV). A brief description of the adsorption-induced dislocation emission (AIDE) is also included. We also give an outlook on the occurrence of the combination of two or more mechanisms.

### Hydrogen-induced decohesion

#### Description

The hydrogen-induced decohesion (HID)^2^ theory hypothesises that there is a reduction in the bonding energy between atoms due to the presence of hydrogen, which consequently increases the risk of decohesion. This was postulated by Pfeil [162] at the beginning of last century by observing the appearance of the brittle fracture surface of both single-crystal and poly-crystalline iron charged with hydrogen.

The HID mechanisms was initially proposed by Gerberich et al. [60] in the 1970s to explain the observed increase in crack-tip-opening angle, which is a consequence of reduced cleavage toughness with increasing hydrogen content. Oriani proposed that hydrogen surpasses the solubility limit within the lattice due to its dilation as a consequence of the elastic-hydrostatic stresses. Further studies on the HID phenomenon identified trapping sites as a location for hydrogen segregation, such as grain boundaries, reducing the cohesive strength locally between metal atoms. HID states that hydrogen embrittlement occurs within the crack tip fracture process zone, where the tensile stress exceeds the maximum-local atomic cohesive strength, which, as stated, is reduced in the presence of hydrogen.

The HID mechanism has been used to explain the brittle intergranular fracture surface observed in high-strength steels; however, it should be noted that hydrogen-induced decohesion remains unproven by direct experimental methods.

#### Evidence supporting the HID mechanism

**Figure 2:**
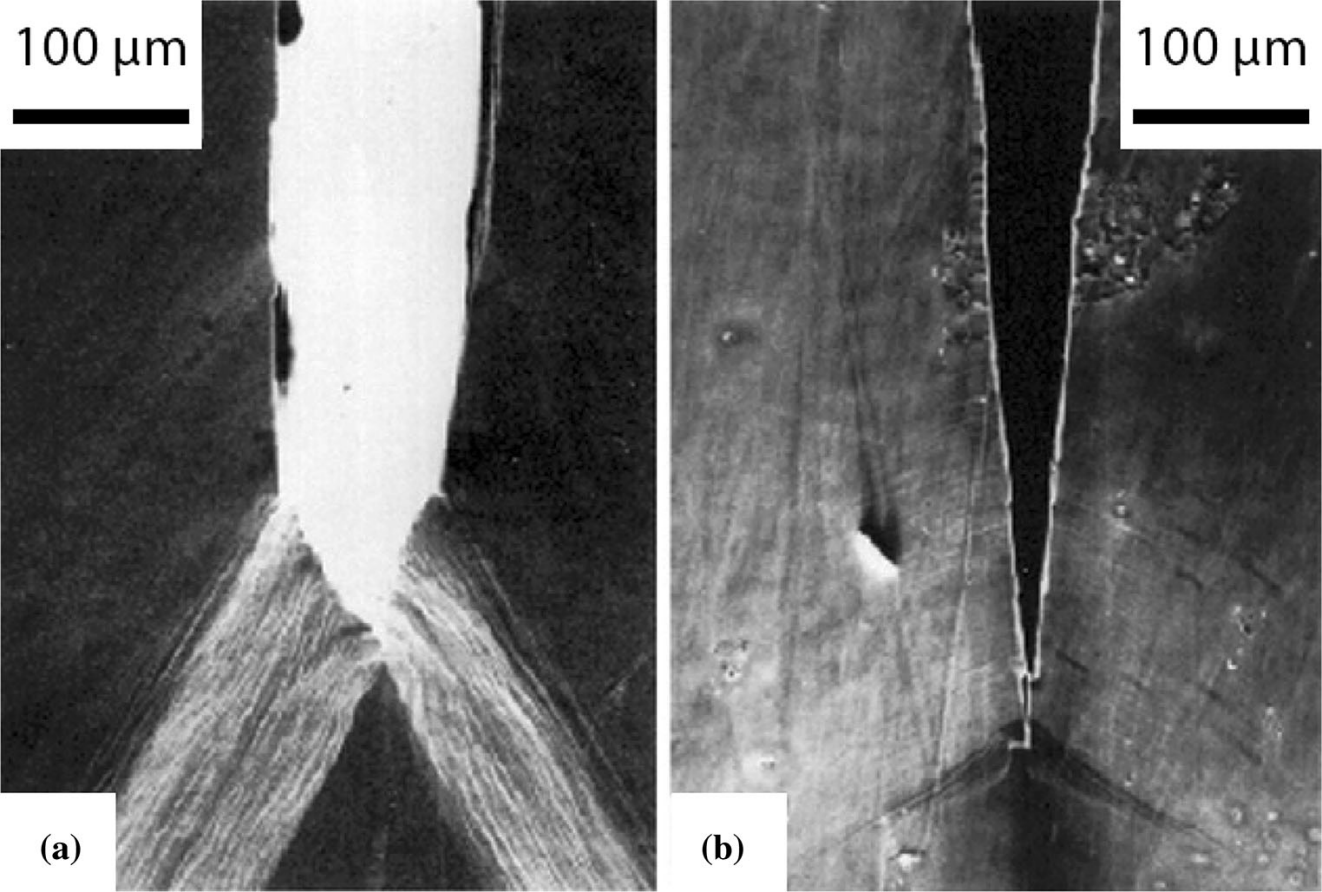
Crack-tip-opening angles of a Fe–Si (3 wt%) single crystal after straining in **a** a vacuum and **b** hydrogen [6]

**Figure 3:**
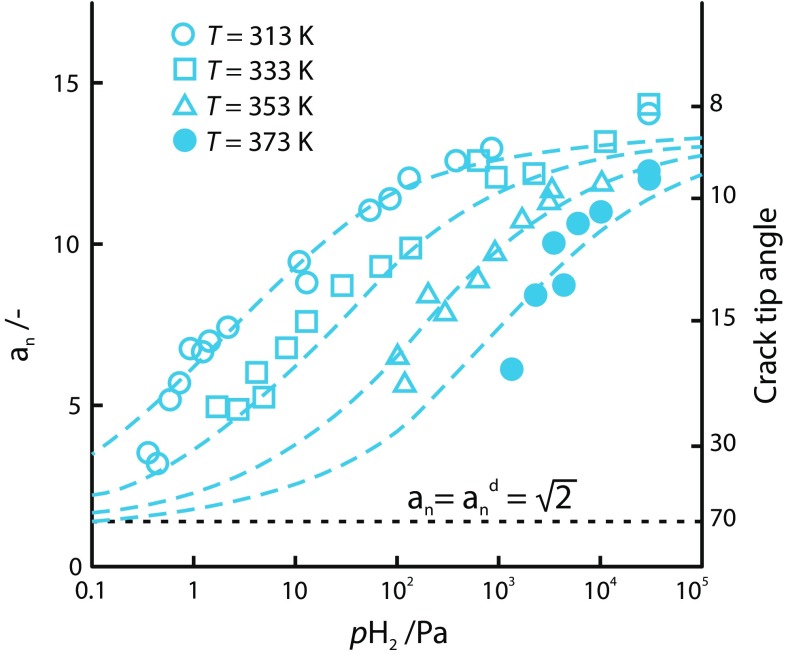
Crack-tip-opening angle as a function of in situ hydrogen pressure $(P_{\mathrm{H}_{2}})$ for a Fe–Si (3 wt%) single crystal over a range of temperatures. The horizontal dashed line indicates crack growth solely by crack tip slip. Figure adapted from [221]

The HID mechanism is supported by the fact that hydrogen tends to diffuse to areas of high stress, similar to that found ahead of a crack tip. Furthermore, the density of the hydrogen trapping sites increases along a crack path [154]. Atomistic simulations have also validated the hypothesis of the reduction in atomic cohesion with increasing hydrogen content. However, the effect of hydrogen on atomic cohesion cannot be validated experimentally [36]. The predominant experimental proof for HID is in the observed reduction in the crack-tip-opening angle with increasing hydrogen content as shown in Fig. 2 [220]. Figure 3 shows the dependence of in situ crack-tip-opening angle on both hydrogen pressure and temperature for a 3 wt% Si-doped iron single crystal, indicating that as decohesion-based crack growth becomes increasingly important, the angle between active slip planes is reduced. This implies that since the crack planes of the samples were parallel to (100) and no dimples were observed on the fracture surface, the decohesion mechanism becomes more dominant, replacing the crack tip slip mechanism.

A number of models based on the HID theory have been developed in recent decades using both dislocation mechanics and fundamental continuum fracture mechanics. These models have proved to be effective in emulating experimental results for fracture toughness and crack growth rates [26, 60]. The work in [26, 60] focuses on concurrent dislocation activity and interfacial decohesion by bond breaking in iron and steels. Jokl et al. [87] proposed a thermodynamic fracture criterion based on Irwen–Orowan–Griffith theory applicable to crystalline solids which is capable of being plastically deformed. Their theory is based on the experimental fact that during the propagation of a brittle crack, the energy is used not only for bond breaking but also for dislocation emission from the crack tip. To formulate their criterion they also use an empirical relation between stress and dislocation velocity to calculate work connected with dislocation emission. Liang and Sofronis [115] studied the role of hydrogen on the initiation of and propagation of interfacial debonding coupled with material plasticity. They also studied a potential competitive or synergetic action between HID and material softening in promoting void nucleation or plastic localisation. At the cutting edge of research aimed at legitimising HID is the desire to demonstrate the effect of the dissolved atomic hydrogen on (a) lowering the interatomic force–displacement relationship in metals and the resultant effects on surface energies, (b) the material properties that induce the observed changes in the mechanical behaviour. The design of an appropriate experiment to resolve these issues has proven difficult due to the high concentration of hydrogen within the fracture process zone and the difficulty of reproducing this content within a characterisable bulk specimen, due to the limited hydrogen solubility within the bulk [5]. As modelling techniques and most notably computational power improves, it is viable that a deeper insight into hydrogen’s effects on bonding may be achieved in the near future. However, the occurrence of the HID mechanism does not illegitimise other theories of hydrogen embrittlement.

#### Atomistic simulations supporting the HID mechanism

The primary method to demonstrate the HID mechanism with atomistic simulation involves calculating a lowering of the energy required to separate grain boundaries. Recent reviews of the field have highlighted the studies carried out using empirical potentials [178]. These parameterised models for interatomic interactions are fast enough that large grain boundary systems of thousands of atoms can be simulated using molecular dynamics; however, their predictive ability is limited by the accuracy of the potential.

Wang et al. modelled the interaction of hydrogen using an empirical embedded atom model potential (EAM), across a wide range of grain boundaries that span the complete range of misorientation angles [229]. By identifying the optimum adsorption sites at the grain boundaries for all hydrogen atoms, at various external hydrogen concentrations, and optimising the trapping of hydrogen at the free surfaces produced by separating each grain boundary, they predict a 37% drop in the cohesive energy at conditions known to give intergranular fracture experimentally, supporting a hydrogen-induced contribution to decohesion.

Molecular dynamics of dislocations interacting with grain boundaries, also carried out using an EAM, demonstrate that the presence of hydrogen at the grain boundaries increases dislocation density and build up of strain [1]. The release of the strain is postulated as a mechanism for a transition to intergranular fracture. However, the findings in [229] and [1] show that the HID mechanism is not sufficient to explain the formation of intergranular fracture; indeed, they enforce the contribution by local plasticity.

Even where the most accurate interatomic potentials have been trained to reproduce excellent hydrogen dissolution energies [171], there are trade-offs, for example, in the accuracy of binding energies at surfaces, which is an essential component of grain boundary based decohesion calculations. The EAMs have also been shown to reproduce general trends in grain boundaries, but do not always reproduce the ground state structure and energy found with ab initio DFT methods [185]. DFT will give the most accurate description of the interaction of hydrogen with grain boundaries; however, the computationally expensive quantum mechanical treatment of the system means that grain boundary systems are limited to around a hundred atoms compared to thousands that can be simulated with empirical potentials.

A number of studies based on DFT calculations are able to provide compelling evidence for the decohesion mechanism:

Du et al. [41] carried out a DFT study of hydrogen interactions at grain boundaries of BCC and FCC iron. On both close-packed $\Sigma$3-type and more open $\Sigma$5-type grain boundaries of BCC iron, the optimal solution energy of hydrogen can be as low as $-\,0.18$ eV (where a negative value indicates solution is preferred), compared to 0.25 eV for the bulk solution. By application of Griffith’s criterion (which approximates the decohesion by comparing fractured and non fractured systems) they find that trapped hydrogen facilitates fracture along all of the grain boundaries, compared to clean surfaces. An analysis of the hydrogen diffusion pathways shows that the trapped hydrogen impedes the diffusion of incoming hydrogen which could account for the experimentally observed strain rate dependence of the ductile to brittle transition.

Further research by Momida et al. [134] investigated the effects of vacancies at $\Sigma$3-type grain boundaries on hydrogen embrittlement. Hydrogen is known to stabilise vacancies in the bulk [70]. In a series of models of grain boundaries, both with and without vacancy defects, the results of Du et al. [41] were confirmed, i.e. increasing hydrogen concentrations lowered the tensile strength of the grain boundary. The embrittlement effect was modelled under static strain by using a model where pinned atoms were pulled apart whilst measuring the effect on the energy. The resultant stress–strain curves quantify the reduction in tensile strength and the work required to separate the grain boundaries. Although trapped hydrogen was confirmed to enhance fracture, vacancy defects were found to prefer sites closer to the grain boundaries where they aggregate hydrogen and the decrease in tensile strength caused by the hydrogen vacancy complexes is greater than combination of the separate effects. In this case, the delayed transition to brittle fracture is attributed to the low mobility of vacancies that need to aggregate at the boundaries for embrittlement.

Tahir et al. [202] carried out similar simulations in which they calculated the work of separation of $\Sigma$5-type grain boundaries in which they also included interstitial carbon. Their results show that carbon is fully segregated at the grain boundary surface in the ground state, giving a 15% increase in the work of separation. Co-doping with carbon and hydrogen results in an 18% reduction in the work of separation. The reduction in the work of separation increases as more carbon is displaced by hydrogen, demonstrating the mechanism of embrittlement. In this case the delay of brittle fracture can be attributed to the rate at which hydrogen is able to displace segregated carbon.

Although these studies provide support for various mechanisms of decohesion, there are still open questions before a consensus can be reached on which is most important in real systems. One important challenge will be the convergence of the results from different simulation methods for grain boundaries with higher misorientations, which have been shown to be responsible for embrittlement in other metals [14]. Although the simulations with empirical potentials support decohesion across a range of misorientation angles [229], the quantitative behaviour will need to be validated by DFT to ensure the accuracy of the results. This is a challenge as high misorientation boundaries require larger simulation sizes, but the computational expense for DFT scales with the cube of the number of atoms. Another solution to this problem would be to develop a new generation of interatomic atomistic or multiscale models that correctly reproduce the interaction of hydrogen with grain boundaries during fracture and can be used in systems that model thousands of atoms.

#### Mesoscopic models of the HID mechanism

Modelling approaches at the mesoscale have usually been postulated to estimate the decay in mechanical properties produced by the hydrogen-induced decohesion mechanism. For instance, many semiempirical models describing the variation in the fracture stress under the presence of hydrogen have been proposed. Zinbi and Bouchou [244] have qualitatively explained how hydrogen can cause brittle fracture via decohesion. In the absence of hydrogen the fracture stress $\sigma _{\mathrm{f}}$ necessary to cause the propagation of an elliptical crack of length 2*l* is $\sigma _{\mathrm{f}} = \sqrt{\frac{2E\gamma _{e}}{\pi l}}$, where *E* is Young’s modulus and $\gamma _{e}$ is the surface energy of the material. When hydrogen is absorbed, it decreases the local bond strength and surface energy. The reduction in fracture strength can be expressed as a function of hydrogen concentration in the bulk $C_{\mathrm{l}}$ by defining the equivalent fracture strength of a material containing hydrogen $\sigma _{{\mathrm{f}},{\mathrm{H}}}$ as follows:

9 $$\begin{aligned} \sigma _{{\mathrm{f}},{\mathrm{H}}}=\sigma _{\mathrm{f}}-\beta C_{\mathrm{l}}^\eta \end{aligned}$$

where $\beta$ and $\eta$ are fitting parameters. This equation represents the effect of hydrogen diffusing to a crack tip and assisting in further propagation. Additionally, Ohata et al. [151] have used a semiempirical expression for $\sigma _{{\mathrm{f}},{\mathrm{H}}}$ that was obtained by the weakest link theory. They tested the model for different loading and H concentrations, showing that local stress concentrations are not only influenced by the diffusible hydrogen but also by the geometry of the components.

Novak et al. [150] have proposed a physically based statistical micromechanical model for the fracture strength in a medium carbon steel. They modelled the behaviour of an interface crack in a grain boundary carbide ahead of the notch tip, where carbide particles were considered to be subject to cracking/decohesion along the particle matrix interface. The model considers the synergistic action of both the HID and another mechanism, hydrogen-enhanced local plasticity (HELP), which will be discussed in detail in "Hydrogen-enhanced localised plasticity" section, in dictating failure and the effect of particle size during decohesion.

Indeitsev et al. [80] proposed a model for the fracture stress as a function of hydrogen content based on statistical mechanics. They estimated the number of broken chemical bonds produced by hydrogen $N_{\mathrm{H}}$ using Fermi–Dirac statistics, where the energy is equal to the sum of the plastic energy in the absence of hydrogen and the excess energy induced by hydrogen-induced decohesion. They obtained a formula relating the distribution of hydrogen by chemical bonds $n_{\mathrm{H}}$ and the plastic strain $\varepsilon _{\mathrm{p}}$:

10 $$\begin{aligned} \frac{n_0-n_{\mathrm{H}}}{n_0}=\frac{1}{1+\exp \big (\frac{\varepsilon _{\mathrm{p}}-\varepsilon _0}{\varepsilon _c}\big )} \end{aligned}$$

where $n_0$ is the total number of chemical bonds, $\varepsilon _0$ is the initial strain energy (at $\varepsilon =0$) and $\varepsilon _c$ is a fitting parameter. This equation allows us to evaluate the effective concentration of H involved during decohesion at a given strain. This approach was applied to describe the fracture stress in a pearlitic steel using a semiempirical expression for the stress–strain response of a two-phase material in the presence of hydrogen:

11 $$\begin{aligned} \sigma _{Y}=\frac{E\varepsilon _{\mathrm{p}}}{1+(\frac{E}{E_{\mathrm{H}}}-1\big )\frac{n_{\mathrm{H}}}{n_0}} \end{aligned}$$

where $E_{\mathrm{H}}$ is a fitting parameter. This equation shows that the flow stress ($\sigma _{Y}$) decreases when the concentration of hydrogen increases. They found a good correlation with their experiments, although no information of the steel’s microstructure was considered in their calculations.

Quantitative understanding of the influence of hydrogen under cyclic loading is also of great technological importance. Turnbull [215] has made a comprehensive review of available phenomenological models for environmental fatigue crack propagation by hydrogen embrittlement. Although the underlying mechanisms of each model are distinct, the evolution equations for crack growth are usually expressed as the sum of two contributions: (a) the growth rate in the absence of hydrogen and (b) the growth rate associated with the cyclic plastic deformation and the operating embrittlement mechanism. In almost all models, (b) is proportional to hydrogen concentration and local diffusivity.

Xing et al. [233] have proposed a unified model for crack propagation under cyclic loading based on a decohesion mechanism that recovers the form of the Paris Law:

12 $$\begin{aligned} \frac{\mathrm{d}a}{\mathrm{d}N}=\Psi \bigg (\bigg (\frac{1+R_K}{1-R_K}\bigg )\frac{\Delta K^2}{(f/f_\mathrm{{crit}})^{m}}\bigg )^n \end{aligned}$$

where $\Psi$ is a constant that depends on hydrogen concentration, $R_K$ is the ratio between the stress intensity factors at minimum and maximum loading, respectively, $K_\mathrm{{min}}/K_{\mathrm{max}}$, $\Delta K=K_{\mathrm{max}}-K_{\mathrm{min}}$, *f* is the loading frequency, $f_{\mathrm{crit}}$ is the loading frequency under which the crack propagation rate would reach a maximum, which depends on hydrogen diffusivity, and *m* and *n* are fitting parameters. Additionally, Holobut [78] has employed a cohesive zone model to describe the increment in the stress intensity factor $K'$ by H-induced decohesion. He postulated that hydrogen induces an additional stress term in $K'$ accelerating decohesion and it depends on how fast H can diffuse to the crack tip. In this case the Forman equation was used to predict the crack propagation rate in terms of $K'$. Similarly, Lu [118] has found that the crack growth rate is inversely proportional to the concentration of hydrogen.

These models are based on a rate-limiting process and usually do not consider the microstructural features controlling the mechanisms for crack initiation and propagation, such as microstructural trapping of hydrogen or explicit microstructure dependence.

#### Continuum models of the HID mechanism

Continuum models of HID often assume that material failure occurs when a critical hydrogen concentration is reached locally [2, 135]; however, this criterion is not based on any well-accepted physics nor has it been proved experimentally. Novak et al. [150] proposed a model of the embrittlement process due to hydrogen charging in martensitic high-strength steels in terms of intergranular fracture. They observe that cracking follows the prior austenite grain boundaries and cracks occur at decohering carbides or second-phase particles. In their model the failure process initiates by decohesion at grain boundary carbide particles and the intensity of the failure event depends on the local stress and hydrogen accumulation associated with a dislocation pile-up at the matrix–carbide interface.

There is enough confidence among researchers to suggest that there might be more than one mechanism occurring simultaneously. However, this has not been proven experimentally. Recently, a model, based on a classical continuum mechanics formalism, which takes into account the synergetic effect of two of the hydrogen mechanisms: HID and HELP (described in "Hydrogen-enhanced localised plasticity" section) has been proposed [7, 8]. Recent evidence from continuum modelling of the coupling between hydrogen diffusion and the mechanical behaviour of metals show that in order to observe embrittlement the HELP mechanism needs to be accompanied by another mechanism such as HID [9].

Experimental observations of the microstructure and fracture morphology of a ${\hbox {M}}_{7}{\hbox {C}}_{3}$-carbide-rich region of a dissimilar AISI8630/IN625 weld reveal that cracks nucleate at the interface of the ${\hbox {M}}_{7} {\hbox {C}}_{3}$ particles in the carbide-rich region under the high stresses generated ahead of a crack tip, followed by strain localisation between the particles linking these interfacial cracks. The model simulates the failure process at the interface of the ${\hbox {M}}_{7} {\hbox {C}}_{3}$ particles and the matrix by use of a cohesive zone model (CZM) whose traction–separation law is a function of hydrogen content and the plastic deformation of the surrounding material. The constitutive response of the matrix (a nickel alloy) is a function of the hydrogen content. The results show that in regions where the hydrogen content is high, the cohesive strength decreases and microcracks form at the particle/matrix interface, leading to microvoid formation. Deformation then becomes localised in the region where (a) the hydrogen content is high and (b) the carbide/matrix interface has debonded. As deformation proceeds, the interfacial microcracks grow and link due to intense localised plastic flow between the carbides. The failure surface consists of planes of intense plastic deformation, connected by small regions of decohesion around the particles, which is consistent with experimental evidence. In the next section a detailed description of the HELP mechanism is presented.

### Hydrogen-enhanced localised plasticity

#### Description

The hydrogen-enhanced localised plasticity (HELP) theory [19] postulates that hydrogen embrittlement is a result of the increased mobility of dislocations due to the presence of hydrogen. This phenomenon might be seen in contradiction to the well-established phenomenon whereby increased dislocation mobility enhances ductility in metals. The HELP theory suggests hydrogen has a shielding effect on the elastic stress field of dislocations, thus enhancing the dislocation mobility and slip localisation [19, 175]. The increased dislocation mobility due to various interactions with the Cottrell-like hydrogen atmospheres surrounding dislocations, and its resulting effects on elastic interactions, is discussed in more detail later.

For FCC metals, a significant amount of evidence exists surrounding the HELP mechanism. The stacking fault energy (SFE) in FCC metals is thought to decrease in the presence of hydrogen. This has been shown through both modelling [119] and experimental work [52]. Experiments have also found that hydrogen promotes slip planarity in FCC metal [175]. The reduced SFE results in an increase in dislocation mobility local to the regions of high stress concentrations, e.g. crack tips, due to the hydrogen accumulation. The decreased SFE was not originally postulated within the HELP mechanism, but the enhanced planar slip, as a possible result of reduced SFE, can contribute to the localised plasticity. Generally, as the SFE decreases, the constriction work of partial dislocations increases, restricting cross-slip. Despite this, there is no consensus on whether a reduction in SFE (typically around 20% [52]) alone is sufficient to affect cross-slip. Regardless, hydrogen has been shown to decrease the repulsive forces between dislocations, thus promoting their mobility, which can then pile-up against barriers, e.g. grain boundaries, carbides. As hydrogen is attracted to the strain fields surrounding a dislocation, it forms a Cottrell atmosphere, which results in a reduced yield strength [5]. It is thought that this effect results from hydrogen screening the elastic field of dislocations. This is the direct opposite of what is observed for carbon-dislocation interactions and the effects of their corresponding Cottrell atmospheres. The reason for this is that carbon has a lower diffusivity than hydrogen. When hydrogen in the atmosphere cannot move with dislocation, i.e. when strain rate is very high, the situation is similar to the carbon-dislocation interactions. Solute drag effect and serration of flow stress are observed.

**Figure 4:**
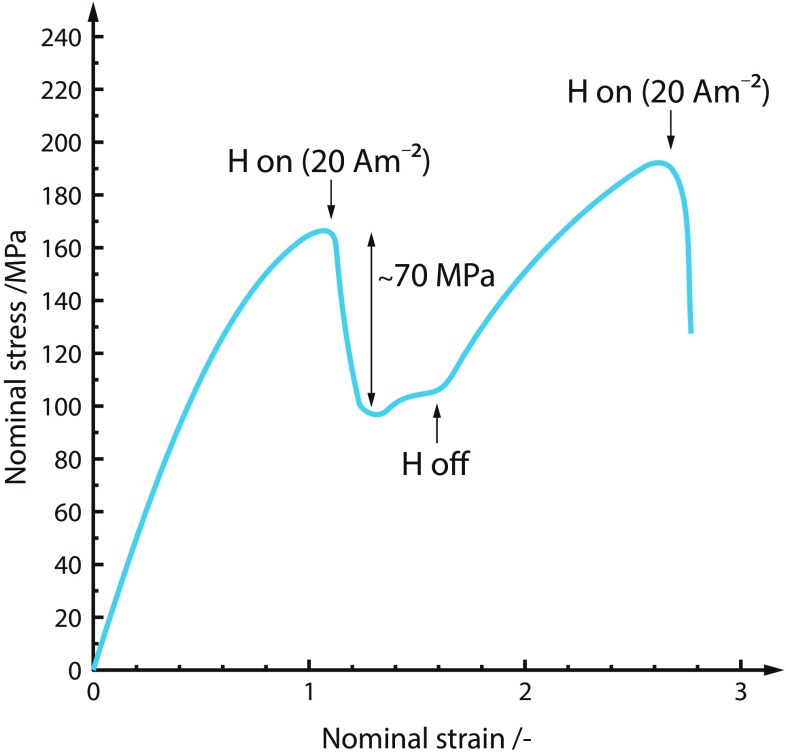
Results from tensile testing of high purity iron at 200 K with a strain rate of $8.3 \times 10^{-5} \hbox {s}^{-1}$ with hydrogen charging ($20 \, {\hbox {Am}}^{-2}$) in $0.1 \, \hbox {N} \hbox {CH}_{3} {\hbox {OH-H}}_{2} {\hbox {O-H}}_{2} {\hbox {SO}}_{4}$ solution switched on and off. Adapted from [84]

Enhanced ductile processes due to a hydrogen interaction had first been suggested by Beachem [13]. Strong evidence for failure by hydrogen-enhanced local plasticity in a range of steels has been obtained by macroscopic flow stress measurements, fractographic evidence, in situ TEM studies, measurements of dislocation motion and theoretical treatments [67, 69, 107, 111, 176, 191, 237]. Such enhanced plasticity, resulting in localised plastic failure, suggests that hydrogen promotes shear decohesion along slip planes in contrast to the usual sense of embrittlement. The result of HELP is that less stress is required to induce a given plastic strain. Consequently, this phenomenon has been labelled as the softening effect [190], an example of which is shown in Fig. 4. In the authors’ opinion softening of the constitutive response due to the presence of hydrogen does not lead to localisation of strain and a macroscopic brittle response [9] as also mentioned in "Continuum models of the HID mechanism" section. Softening must be combined with other degradation process for the material to embrittle. However, when it is believed that HELP is one of the underlying mechanisms, fracture can be either intergranular or transgranular, depending upon the microstructure and hydrogen concentration distribution [19, 52].

#### Evidence supporting the HELP mechanism

There are numerous studies validating the HELP mechanism in various steels using various testing and characterisation techniques [52, 67, 69, 107, 111, 176, 191, 196, 237]. As such, the key papers from which the HELP theory has been developed and that provide the most conclusive evidence will be summarised. Beachem [13] in the 1970s was one of the first authors to have hypothesised that hydrogen dissolved in the lattice enhances ductility through reducing the threshold stress to unlock dislocations, i.e. in the presence of hydrogen dislocations can move at a reduced stress. This phenomenon leads to severe plastic deformation to a level at which macroscopically brittle sub-critical crack growth occurs.

**Figure 5:**
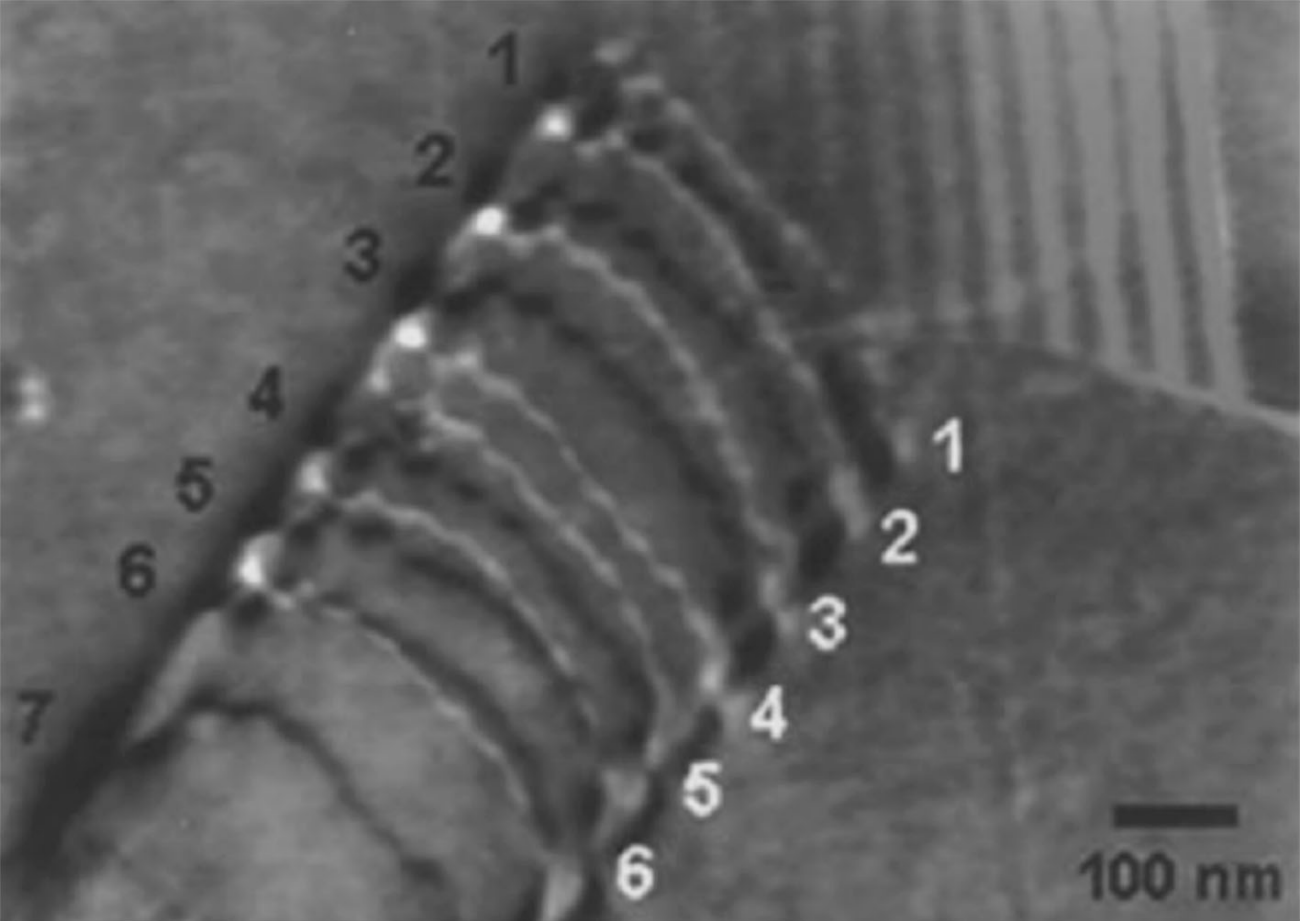
Superimposition of two TEM images of a single dislocation pile-up upon a grain boundary under constant stress; one within a vacuum (black), the other under 95 Torr of hydrogen gas (white) in 310 S stainless steel [52]

Ferreira et al. [52] evaluated the interactions between dislocations in austenitic stainless steel (310 S) by using in situ hydrogen charging within an environmental TEM. The effect of hydrogen in solution screened the elastic interactions between perfect and partial dislocations and any obstacles to dislocation motion. The observed result was an increase in the dislocation mobility. This results in an increased density of dislocation pile-ups at interfaces such as particles or grain boundaries as shown in Fig. 5. The initial dislocation configuration was produced by deforming the sample in a vacuum. Hydrogen gas was added to the TEM cell, and the induced increase in pressure was seen to cause a reduction in the equilibrium distance between dislocations at the grain boundary, as shown in Fig. 5. It should be noted that the in situ observations during hydrogen pressure increase were carried out on very thin films, with no plastic constraint, and the dislocations observed are only those pinned on both sides of the film. Thus, there are drawbacks to using TEM-based hydrogen evaluation, most notably its limitation with respect to simulating real conditions. Worthy of note, Ferreira et al. also discovered that upon the introduction of hydrogen into the TEM chamber, stationary cracks began to propagate both along grain boundaries in the presence of impurities such as sulphur, and within the adjacent matrix. Although Ferreira et al. [52] cite that free hydrogen in solid solution reduces the critical stress for dislocation movement by preventing dislocations from interacting with elastic obstacles, another study published by the same authors [51] and some more recent studies [111, 237] have shown that HELP is due to reductions in the stacking fault energy, reducing susceptibility to cross-slip by increasing the equilibrium distance between partial dislocations. This increased ductility is suggested to cause the localised softening, increasing plastic failure susceptibility. Consequently, this phenomenon has been labelled the "softening effect", contrary to the typical perception of embrittlement.

#### Atomistic and mesoscopic models of HELP

The validity of the constitutive assumptions employed by continuum models describing hydrogen-metal interactions can be established only to the extent that the observed behaviour is not contrary to direct experimental evidence. A comprehensive understanding of the HELP mechanism requires a more detailed evaluation than can be provided by phenomenology. Some progress can be made by incorporating information from more accurate atomistic calculations at smaller length scales in continuum models. Fully discrete simulations using empirical potentials have also been used to model the interaction of H with extended lattice defects (general grain boundaries, dislocations, cracks) but the predictive power of these studies is, to a great extent, limited by the reliability of the interatomic potential. The first-principles calculations are mostly limited to studies of bulk phases, point defects, high-index surfaces and simple grain boundaries.

The influence of plastic deformation on H transport is generally associated with the competing roles of dislocations as trap sites or vehicles for hydrogen transport. Studying H migration and interaction in the presence of microstructural imperfections requires simulations in large blocks of atoms for simulation times exceeding the typical atomistic timescales. Therefore, direct simulations of rare events, such as thermally activated processes, are excluded by this approach. The kinetic Monte Carlo (kMC) method has the advantage of being computationally less expensive because the interatomic interactions are not computed during the simulation. Instead, kMC uses precomputed transition rates along the minimal energy paths between the metastable sites, thereby allowing employment of more precise electronic structure methods. Ramasubramaniam et al. [170] have developed an off-lattice, on-the-fly kMC model for simulating stress-assisted diffusion and trapping of hydrogen by crystalline defects in BCC iron. The model employs precomputed transition rates obtained with high-accuracy DFT calculations in defect-free parts of the crystal. The energy barriers to diffusion on a sphere of atoms surrounding the diffusing H atom are computed on-the-fly by using an EAM potential. The results from kMC simulations have been found to be in good agreement with trapping theory for trapping at screw dipoles. The kMC method has been also employed by Du et al. [42] to investigate hydrogen diffusion in BCC iron within microstructures representing different arrangements of grain boundaries and point defects. All input data to the kMC model are obtained from first-principles calculations. It has been found that the overall hydrogen diffusivity within the interface region is lower than in pure BCC Fe crystals.

To reach the length- and timescales relevant for engineering applications, Leyson et al. [114] have developed a multiscale model that can combine the resolution of atomistic effects with the computational efficiency associated with continuum models. The model takes as input quantities that are computable by atomistic calculations and is thus able to take into account atomistic effects. Due to atomistic inputs, the model contains all the essential features of atomic interactions. It can faithfully reproduce hydrogen–hydrogen interactions and dislocation core structures due to the localisation of hydrogen around the dislocation. The computational efficiency of the model makes the calculation of the equilibrium concentration profile of hydrogen, normally inaccessible to direct atomistic calculations due to timescale constraints, attainable. The analytic nature of the model allows for the simulation of large defect structures, such as dislocation pile-ups and dislocation loops. It can serve as an important step towards studying the effect of hydrogen localisation around dislocations on the HELP mechanism.

An efficient approach to address the problem of a large interval of relevant length- and timescales inherent in the study of the effect of H on dislocation–dislocation interactions in FCC metals has been proposed in [226]. The stress-shielding effect has been studied by a multiscale approach combining DFT electronic structure calculations, semiempirical EAM potentials and a lattice gas Hamiltonian and MC (Monte Carlo) sampling. The H distribution around edge dislocations is found to depend critically on H–H interactions. Even weak H–H interactions dramatically reduce hydrogen bulk concentrations required to induce the stress-shielding effect underlying the HELP mechanism. The stress-shielding effect is correlated with a reduced dislocation separation at dislocation pile-up tips, which may result in the nucleation of microcracks. As a consequence, low H bulk concentrations may result in embrittlement.

Plasticity in BCC metals is mainly mediated by the thermal activation of kink pairs in screw dislocation lines. A possible cause of HE in the HELP mechanism is the softening of materials by H solute atoms owing to increased screw dislocation velocities and reduced flow stress when H atoms are introduced into a BCC metal. If concentrated H atoms induce local slip, the increase in local strain and dislocation density leads to further concentrations of H atoms, followed by plastic instability and ductile fracture. Since the mobility of a dislocation in BCC metals is mainly determined by that of the screw component, investigation of the interaction between a screw dislocation and an H atom plays a key role in understanding the HELP mechanism. The accurate determination of the kink-pair formation enthalpy is indispensable for development of a reliable model of dislocation mobility in BCC metals.

Kirchheim [101–104] considered H embrittlement based on enhanced local plasticity in the light of a defactant concept , describing solute–defect interactions in a thermodynamic framework. According to this concept, solute atoms trapped in defects, such as vacancies, dislocations and kinks in dislocation lines, lower the defect formation energy. The general approach proposed by Kirchheim [104] describes local plasticity caused by a lower energy barrier for the generation of dislocation loops in the presence of hydrogen. It is based on a general equation describing the reduction in the defect formation energy by the action of H atoms segregating to them. The defactant concept applied to nanoindentation reveals a lower energy barrier for the generation of dislocation loops in the presence of hydrogen [104]. Within the defactant concept, solute atoms affect both kink formation and kink motion at screw dislocations [105]. Hydrogen decreases the kink-pair formation energy leading to solid solution softening. The high hydrogen diffusivity leads to a slight increase in the activation energy for kink motion, which is retarded by solute drag. The transition from softening to hardening with increasing hydrogen concentration can be explained by a change of the rate of dislocation motion, which at high H concentration is determined by kink motion [105].

**Figure 6:**
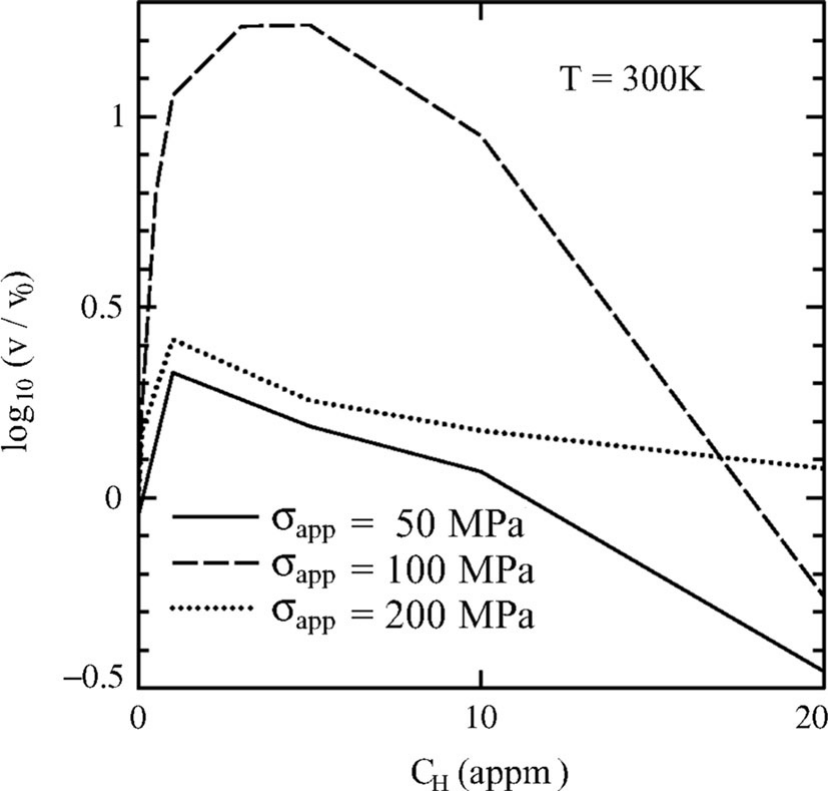
Average velocity of 1/2[111] screw dislocation at different H concentrations and applied shear stresses. At applied stress of 100 MPa and in relatively narrow interval of H concentrations (between 1 and 10 appm) the average dislocation velocity increases by more than an order of magnitude [93, 95]

In several cases the properties of dislocation kink pairs have been determined using a line tension model of a dislocation, incorporating first-principles calculation results [82, 167, 223]. The method consists of properly adjusting the parameters of a one-dimensional line tension model of a dislocation from atomistic calculations performed in small simulation cells. This model is applied to BCC iron to determine the kink-pair formation enthalpy at different applied stresses. The effect of hydrogen on the mobility of a screw dislocation in BCC iron has been investigated by Itakura et al. [83]. A line tension model of a curved dislocation has been employed to elucidate the effect of hydrogen on the kink-pair nucleation enthalpy, kink nucleation rate and dislocation migration process. The interaction energy between a screw dislocation and hydrogen atoms, calculated using first-principles for various hydrogen positions and dislocation configurations, are incorporated into the line tension model of a dislocation line. Using this model, it was found that hydrogen lowers the Peierls barrier when an H atom is trapped ahead of the screw dislocation on the slip plane as shown in Fig. 6. An H atom trapped behind the dislocation line can slow or stop the kink motion and decrease the dislocation mobility. The softening effect of H atoms by promoting kink nucleation and the hardening effect by impeding the kink movement were both evaluated. Four conditions for the overall softening, consisting of two upper critical stresses and upper/lower critical temperatures, were derived and critical temperature for transition between softening and hardening behaviour is predicted. Narayanan et al. [143] have developed an atomistically informed crystal plasticity model for studying a broad class of BCC metals with the strength/rate-limiting mechanism controlled by thermally activated dislocation motion via kink nucleation. The crystal plasticity flow rule is informed by an atomistically computed stress-dependent kink-pair activation energy. The nudged elastic band method is used to capture the minimal energy pathway and evaluate the stress dependent activation parameters of kink nucleation. The constitutive model quantitatively predicts temperature and strain rate dependencies of the yield stress, both of which agree with experimental results in the 200–350 K temperature regime.

#### Continuum models of HELP

At the continuum level, supportive arguments for the hydrogen-induced slip localisation have been provided by Birnbaum et al. [19]. These include theoretical findings related to dislocation mobility, shear localisation and elastic shielding of dislocations. Theoretical investigations have been carried out in an effort to understand how the underlying mechanism for hydrogen-enhanced dislocation mobility can lead to macroscopic shear localisation or a necking bifurcation. The reasons for the enhanced dislocation motion due to hydrogen are not yet established. One mechanism which was studied by Robertson and Birnbaum [176] is based on "elastic shielding" of dislocations by hydrogen atmospheres. In this mechanism the mobility of hydrogen allows it to diffuse to the lowest energy sites and form atmospheres around dislocations. Finite element calculations were carried out to study the hydrogen atmosphere configurations and the interaction energies. It has been shown that this shielding effect can account for the observed hydrogen-enhanced dislocation mobility [176]. A very important aspect of hydrogen embrittlement is that there exists a transport stage [75] of hydrogen to the sites where degradation occurs. H transport is significantly affected by plastic deformation, which can either enhance or impair H transport depending on the loading conditions and the inherent H diffusivity of the metal. It has been proposed that H interactions with dislocations, and the formation of H atmospheres around dislocations is a very important aspect of hydrogen embrittlement and its significance lies behind the embrittling mechanisms [75, 176]. Several approaches to modelling the effect of trapping on hydrogen transport have been proposed by Oriani [153], McNabb and Foster [128] and Sofronis and McMeeking [193] as described in "Experimental techniques to detect hydrogen in metals" section. Sofronis and coworkers [116, 120, 193, 201] have studied extensively the coupling of nonlinear diffusion phenomena with elasto-plastic deformation. The elasto-plastic material response in the presence of hydrogen is based on classical J2-flow theory. The constitutive model has been modified to account for experimentally observed effects of hydrogen on plastic deformation resulting from hydrogen-enhanced dislocation mobility at the microscale. Sofronis et al. [194] have proposed an empirical model to estimate the flow stress ($\sigma _Y$) under the presence of hydrogen:

13 $$\begin{aligned} {{\sigma }_{Y}}=\sigma _{0}^\mathrm{{H}}{{\left( 1+\frac{{{\varepsilon }^\mathrm{{p}}}}{{{\varepsilon }_{0}}} \right) }^r} \end{aligned}$$

where

14 $$\begin{aligned} \sigma _{0}^\mathrm{{H}}=\Omega \left( {{C}} \right) {{\sigma }_{0}} \end{aligned}$$

where $\sigma _{0}^{\mathrm{H}}$ is the initial yield strength in the presence of hydrogen, which decreases with increasing hydrogen concentration. $\Omega (C)$ is a monotonically decreasing function of hydrogen concentration. $\sigma _{0}$ is a reference stress and *r* is a material parameter.

The authors [194] highlighted that: "the proposed model in Eqs. 13 and  14 for the hydrogen-induced material softening should not be interpreted as a precise and exhaustive description of the experimental findings. Rather, the model should be viewed as an attempt to quantify the experimental understanding of the effect of hydrogen on dislocation mobility in a continuum sense".

Liang et al. [116] employed this equation and finite element modelling to study shear localisation and necking instabilities for different hydrogen concentrations. Their model predicts that hydrogen accelerates the deformation modes taking place in the H-free material and promotes localised shear band formation. However, recent findings on modelling of the HELP mechanism by using Eqs. 13 and  14 fully coupled with hydrogen transport in plane strain components, which contains deep and sharp doubled-edged notches, show that softening of the constitutive response due to the presence of hydrogen, does not lead to localisation of strain and a macroscopic brittle response [9]. It has been observed that hydrogen accumulates in high local hydrostatic stress regions; however, high hydrogen concentration results in local softening, i.e. a low yield strength, and therefore a low hydrostatic stress. This is a self-regulated process where a balance between hydrostatic stress, hydrogen concentration and yield strength is achieved. In order to observe embrittlement, the HELP mechanism needs to be accompanied by another mechanism such as HID. Burnish and Vehoff [6] used the thermodynamic analysis proposed by Kirchheim [104] to explain the change in the formation energy of a dislocation loop during nano indentation. The energy of this process is determined by the line tension of a newly formed circular loop, the stacking fault energy of a partial and the work required to extend the dislocation loop. Hydrogen affects the line tension and the stacking fault energy, reducing the energy for nucleation.

Within continuum models, plasticity and stress-assisted diffusion are treated at a phenomenological level. Models that have a more micromechanical basis have been proposed to analyse the hydrogen–plasticity interactions [37, 91]. Such models are based on discrete dislocation modelling. The interactions between hydrogen and mobile dislocations and stress-assisted diffusion are treated phenomenologically. Discrete dislocation modelling has been used to study the problem of crack hydrogen–dislocation interactions. Numerical simulation results show that hydrogen segregation and softening favours formation of dense dislocation pile-ups ahead of a crack. The equilibrium configuration of a dislocation pile-up promotes stress concentrations against microstructural obstacles and formation of facet sized cracks. Diffusing hydrogen lowers the cohesion energy of the facet sized cracks and promotes periodic microfractures along the slip planes.

### Hydrogen-induced phase transformation

#### Description

FCC alloys such as austenitic stainless steels subjected to hydrogen charging show that a large supersaturation of hydrogen can create significant structural changes in the surface layer of the specimens. This phenomenon is known as hydrogen-induced phase transformation (HIPT). There are two types of phase transformations: (a) hydride formation and (b) hydrogen-induced martensitic transformation.

- Hydride forming materials (such as zirconium, titanium, tantalum and other transition metals) experience cracking when the hydrogen content exceeds the solubility limit [7]. This phenomenon is due to the fact that highly brittle hydride precipitates form, resulting in a "low energy" fracture path. There is no controversy in the literature about the hydride formation mechanism. Hydride formation and cleavage fracture occur in systems where hydrides are stable or can be stabilised by application of stress. Microscopical observations and thermodynamic analysis confirm this process [55, 191]. The FCC hydride phase, called $\gamma _{\mathrm{Hydride}}$, is formed through phase separation in the matrix enriched with hydrogen. The $\gamma _{\mathrm{Hydride}}$ is structurally the same as the $\gamma$ phase but presents a larger lattice parameter because of a higher hydrogen concentration. This phenomenon is observed for instance in Fe–Ni alloys [149].
- Hydrogen-induced martensitic transformations consist of the FCC $\gamma$ phase transforming into (1) an HCP phase i.e. $\epsilon$-martensite and (2) a BCC phase i.e. $\alpha$-martensite.
  1. This phenomenon is observed in austenitic stainless steels and Fe–Mn alloys. In order to clarify the difference in the modes of transformation between the $\gamma$ and $\epsilon$-martensite in the same alloy, the authors in [149] examined the effect of cathodic hydrogen charging on the modes of phase transformation in a ternary alloy ${\hbox {Fe}}_{50}$–${\hbox {Ni}}_{50-x} {\hbox {Mn}}_{x}$ with *x* varying from 0 to 50 at.%. Results show that the ${\hbox {Fe}}_{50}$–${\hbox {Ni}}_{50-x} {\hbox {Mn}}_{x}$ alloy subjected to cathodic hydrogen charging can undergo two types of hydrogen-induced transformations, depending on the alloy composition; the FCC $\gamma$ phase forms for $x =0$–20 and the HCP phase for $x=40$–50. A high manganese composition decreases the inclination to hydride formation and promoting stability of $\epsilon$-martensite. It was observed that there is a range of compositions between $x=27$–33 where no transformation is induced, although the alloys absorb far more hydrogen than those with a higher manganese content [149]. Narita and Birnbaum [144] studied the role of hydrogen on the phase transformation $\gamma$ to BCC $\alpha$ in stainless steels (304 and 310). They performed fracture test on these steels under both an aggressive hydrogen environment (i.e. cathodic charging, stress corrosion environment and high-pressure ${\hbox {H}}_{2}$ gas) and a less aggressive hydrogen environment (i.e. one atmosphere of ${\hbox {H}}_{2}$ gas). They observed that the steels tested in an aggressive environment showed a $\gamma$ to BCC $\alpha$ transformation in front of the crack and most importantly that the fracture occurs through the $\alpha$ phase. In contrast, steels tested in a less aggressive environment showed no formation (or small amount) of $\alpha$ phase near the fracture surface. They concluded that the transition $\gamma$ to BCC $\alpha$ plays a fundamental role in HE of stainless steels and that increasing the $\gamma$ phase stability results in improved resistance to HE. Cathodic charging leads to high local stresses due to strong gradients in hydrogen concentration. The high stresses in cathodic hydrogenated specimens introduce plastic deformation in stable austenitic steels; this is proposed to be the driving force for the $\gamma \rightarrow \epsilon$ phase transition [77].
  2. Metastable austenitic stainless steels such as types 301, 304 and 316 are expected to be much more vulnerable to hydrogen embrittlement when hydrogen charged [22, 23, 45, 46, 68, 76, 180, 192] or in a hydrogen gas environment [57, 66, 157, 160, 187, 198]. The embrittlement of metastable steels is due to their inherent tendency for the $\gamma$-austenite phase to undergo a transition to BCC $\alpha ^{\prime }$-martensite when subject to deformation; this is called strain-induced martensite [16, 22, 23, 76, 160, 161, 186]. This strain-induced $\alpha$-martensite phase exhibits higher diffusivity than the $\gamma$-austenite phase [89, 183], thus providing a fast transportation channel for hydrogen to grain boundaries and other regions of localised stress, such as microcrack where hydrogen can enable embrittlement [66, 160]. The larger the volume fraction of the $\alpha ^{\prime }$-martensite the more sensitive the steel is to hydrogen embrittlement due to continuous networks of martensite allowing fast diffusion through the lattice to embrittlement numerous vulnerable embrittlement sites [242]. Moreover, the deleterious effect of H in $\alpha$-martensite is also related to the decreased solubility of H in $\alpha$-martensite. The role of strain-induced martensite in hydrogen embrittlement needs further clarification due to recent reports that the ductility increases the ductility of austenitic steels, and hydrogen charged suppresses the formation of strain-induced martensite in austenitic steels [99] The hydrogen suppression of the formation of strain-induced martensite is reported to lead to a decrease in the yield strength and tensile strength and also localised brittle fracture [4]. Hydrogen-induced transformation of the $\alpha$-austenite to $\epsilon$-martensite phases.

The $\epsilon$-martensite phase is reported to improve ductility in the presence of hydrogen. There has also been reports of pseudo-hydride phase of both the $\gamma$-austenite and $\epsilon$-martensite which have lattice parameters that are approximately 5$\%$ greater than the hydrogen-free phases, these are denoted as $\gamma \, *$ and $\epsilon \, *$ [131]. These hydrides are reported to form a hard and brittle surface layer, several $\upmu {\hbox {m}}$ with numerous microcracks; it is possible that this layer further accelerates the ingress of hydrogen in austenitic steels [200].

#### Atomistic simulations

Bugaev et al. [24] proposed a theory supported by experiments in which hydrogenation of austenitic steels leads to an increase in concentration of the thermodynamic equilibrium host lattice vacancies. Their theory predicts that the loss of the phase stability is induced by the presence of the vacancies. They observe $\epsilon$-martensite in hydrogen-charged stable austenitic Cr18Ni16Mn10 steel. Moreover, TEM studies show a high density of dislocation loops, which is evidence of the high concentration of vacancies which become unstable when hydrogen leaves the sample and the vacancies coalesce to form planar vacancy discs. In accordance with their theory, gaseous hydrogenation under high external pressure does not induce $\gamma \rightarrow \epsilon$-martensite transformation, although the same hydrogen concentration has been obtained during cathodic charging of this steel, which led to formation of $\epsilon$-martensite.

As mentioned in section “Theoretical evidences of hydrogen kinetics and hydrogen preferred sites”, BCC iron exhibits two high-symmetry sites competing to host interstitial impurities: the tetrahedral T-sites and octahedral O-sites. Atomistic simulations in these systems have been carried out using DFT. T-sites have a larger volume, with a more isotropic stress field, and are usually found to be more favourable hydrogen adsorption sites [85, 159]. Work by Sanchez et al. [184] confirms this for dilute solutions of hydrogen in iron (${\hbox {Fe}}_{16}\hbox {H}$ or more) or in situations where the structure is constrained to cubic distortions, but finds that allowing the unit cell to distort tetragonally for an ${\hbox {Fe}}_{2}\hbox {H}$ structure stabilises the anisotropic stress field of the O-site to a lower energy than the T-site. In these body-centred tetragonal (BCT) distorted regions, with high hydrogen concentrations, the diffusion of hydrogen is ten times faster (via O- and T-sites) than in dilute regions (only T-sites).

In a follow-up to that work [28], the authors considered the Bain path, which is a diffusionless tetragonal phase transformation, also extending to a transformation to HCP:

$$\begin{aligned} {\mathrm{BCC}} (c/a=1.0) \rightarrow {\mathrm{BCT}} \rightarrow {\mathrm{FCC}} (c/a=\sqrt{2}) \rightarrow {\mathrm{FCT}} \rightarrow {\mathrm{HCP}} (c/a=1.6, \gamma =60) \end{aligned}$$

In the pure material, BCC is the global minimum with anti-ferromagnetic FCT (tetragonally distorted FCC) and non-magnetic HCP as metastable states. The addition of O-site hydrogen causes significant changes to the energy landscape. The internal stress drives the transformation along the Bain path; metastable BCT, FCT and HCP states are found, but a sharp minimum for $c/a=1.71$ is the lowest energy structure along the pathway. It is found that abrupt changes in the most stable magnetic state are accompanied by sudden changes in cell volume, but that the volume changes in the presence of interstitial atoms is twice that of the pure iron. It is suggested that these differences could be a mechanism for embrittlement by providing a source of defects.

Although this work clearly shows that octahedral hydrogen interstitials can drive a phase transformation, there are still some inconsistencies in the results when compared with other simulation data and experimental observations. Due to the link between the volume changes and the magnetism, an important next step would be to accurately determine the magnetic states along the Bain path allowing for complex magnetic states with more than two atoms, including non collinear spin states which are important in close-packed iron structures [173].

### Hydrogen-enhanced strain-induced vacancy formation

#### Description

**Figure 7:**
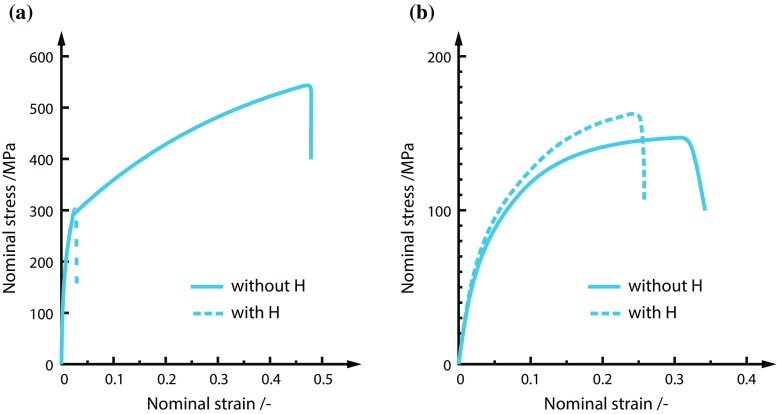
Stress–stress data from tensile testing of hydrogen-charged and uncharged specimens of **a** nickel-based alloy 625, **b** iron. Adapted from [140]

**Figure 8:**
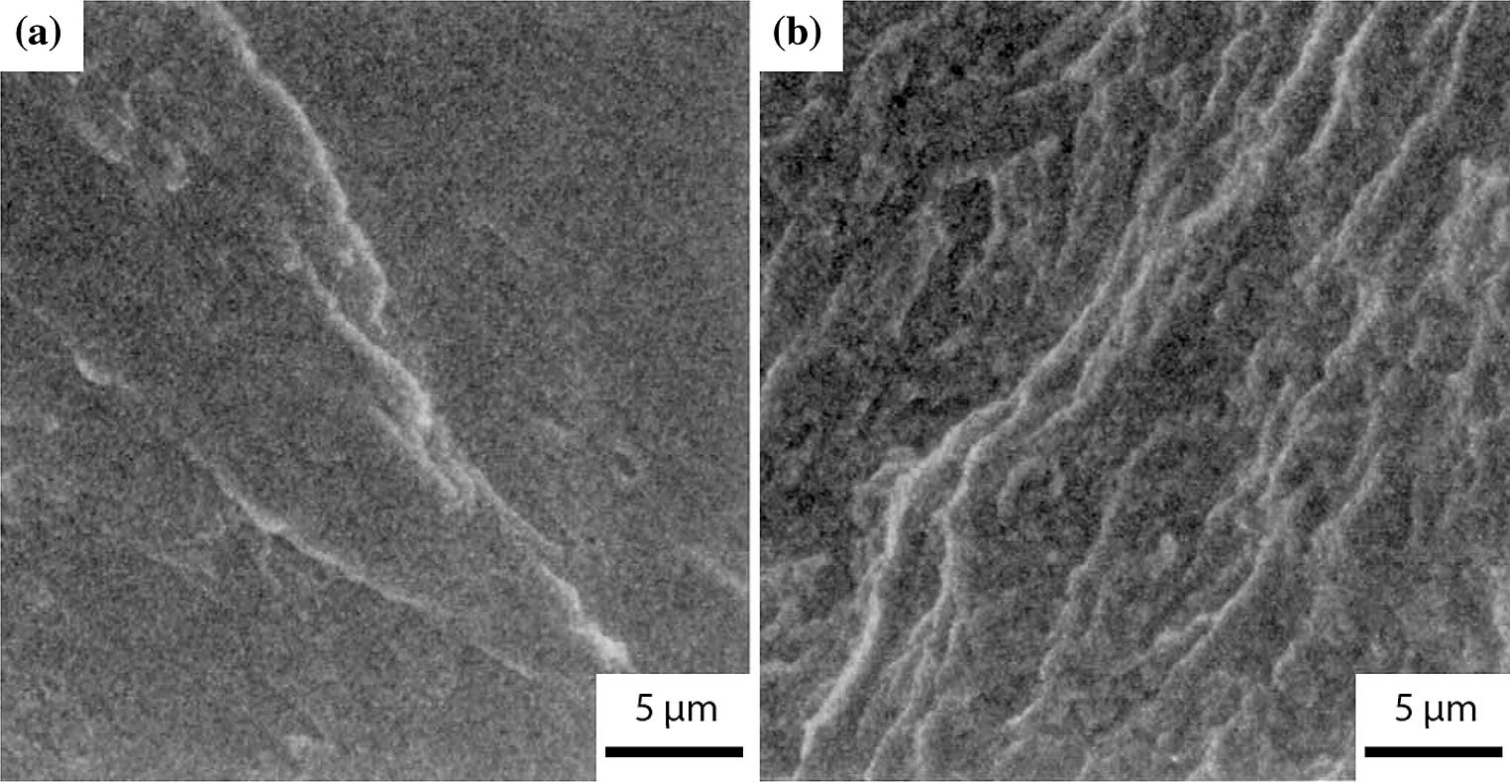
Fracture micrographs from fatigue testing of **a** hydrogen-charged and **b** uncharged $0.57{\mathrm{C}}$–$1.42{\mathrm{Si}}$–$0.65{\mathrm{Mn}}$–$0.67{\mathrm{Cr}}$ steel [142]

**Figure 9:**
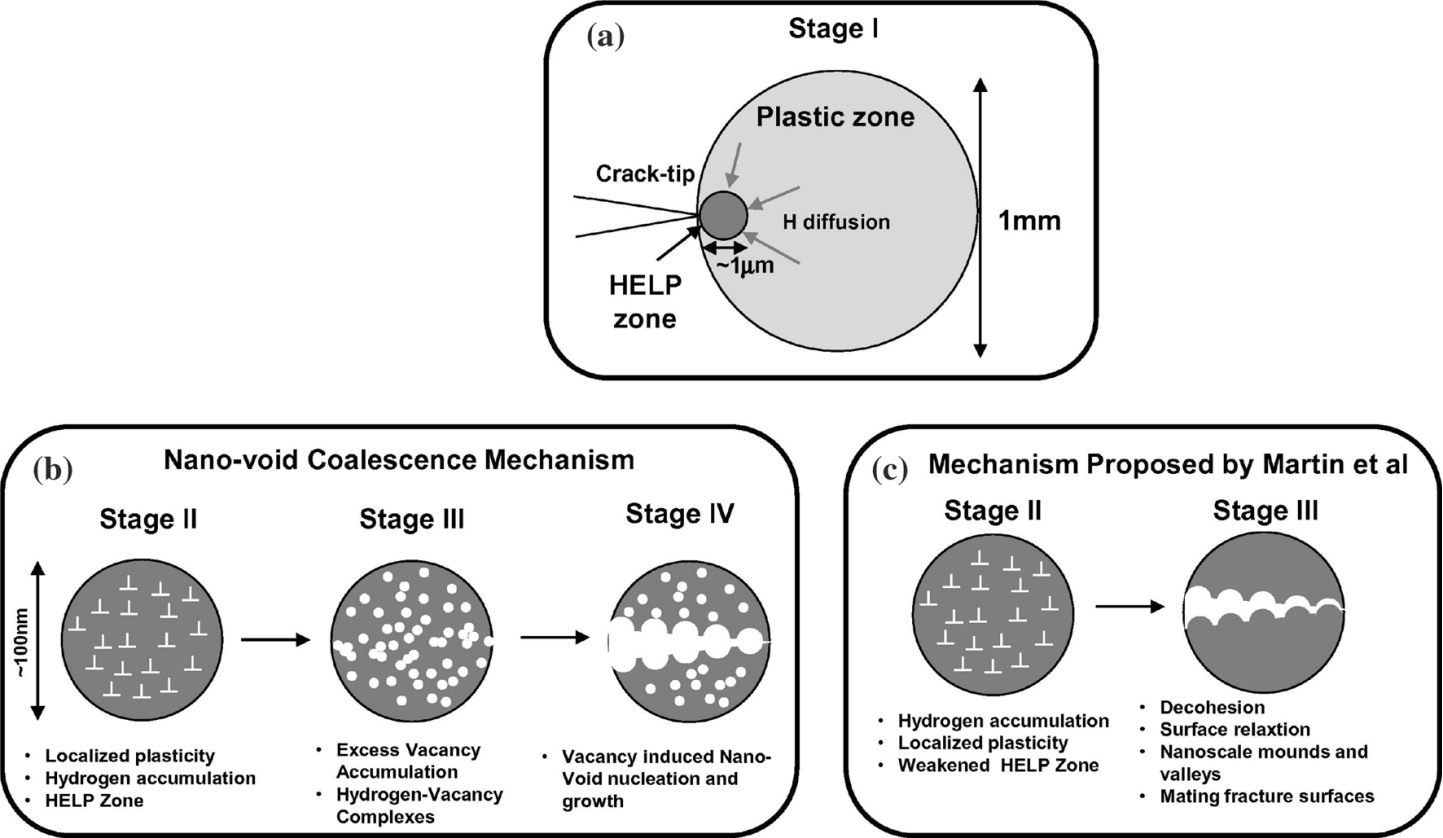
Schematic of macro- and micromechanism [147]

The hydrogen-enhanced strain-induced vacancy (HESIV) formation theory suggests that the density and clustering of vacancies is enhanced in the presence of hydrogen. Vacancies can coalesce to microvoids, which in turn may combine to form larger voids leading to a decrease in ductile crack growth resistance [140]. This phenomenon can be observed during tensile testing and fracture toughness testing of hydrogen-charged samples. HESIV was originally proposed by Nagumo [140], who tested hydrogen-charged nickel-based Alloy 625 and iron, and identified the influence of hydrogen on the stress-strain relationship and fatigue life, as shown in Fig. 7. It was found that hydrogen-charged samples had a higher void density, as predicted by HESIV, after the same fatigue cycles as the uncharged samples. Figure 8 shows fracture micrographs of $0.57{\mathrm{C}}$–$1.42{\mathrm{Si}}$–$0.65{\mathrm{Mn}}$–$0.67{\mathrm{Cr}}$ steel after fatigue testing for both hydrogen-charged and uncharged specimens. In the charged sample (Fig. 8a) the fracture surface appears mainly flat with some irregular ridges showing a quasi-cleavage fracture morphology. In the hydrogen-free specimen (Fig. 8b) the fracture surface appears more ductile. A considerable amount of experimental data further confirms the effect of hydrogen on vacancy formation. Sakaki et al. [182] observed the hydrogen-induced increase in vacancy generation during deformation using positron lifetime measurements. McLellan et al. [127] found that the vacancy density in iron is greatly increased in comparison to that of thermal equilibrium in a high-pressure and high-temperature hydrogen atmosphere, indicating the reduction in formation energy of vacancies due to iron–hydrogen interactions. Vacancies certainly play a role in all of the hydrogen embrittlement mechanisms. Although HESIV alone does not present a conclusive mechanism of embrittlement, Neeraj et al. [147] have proposed a combination mechanism of plasticity-generation (HELP), hydrogen-enhanced vacancy formation (HESIV) and nanovoid coalescence to explain the fracture pathways of both X65 and X80 which failed by quasi-brittle fracture. This is presented pictorially in Fig. 9b.

#### Atomistic simulations

Accurate modelling of the HESIV mechanism requires large systems and long timescales. In order to model the HESIV mechanism, the interactions between cracks and a second-phase microstructure including voids and precipitates needs to be simulated. These systems have been studied with atomistic techniques, although the scope has to date mostly been limited to two dimensional model systems [20]. Examples in $\alpha$-Fe include: interactions of cracks with voids [117], where it was found that voids influence the critical stress intensity factor for dislocation emission from crack tips; interactions of cracks with Cu precipitates [124, 218]; the interaction of cracks with $\gamma$-Fe precipitates [243], and with pre-existing grain boundaries [50, 212].

The interatomic potentials used for iron in all of these studies have been widely criticised for not being sufficiently accurate to be truly predictive, complicating comparison with experiment [133]. Possible resolutions include: (1) improved interatomic potentials, e.g. coarse-grained from electronic structure in the bond order potential formulation [126] or automatically constructed using machine learning (ML) from a database of reference quantum mechanical (QM) configurations [10, 15], (2) concurrent multiscale approaches where localised regions are modelled with QM precision within a non-uniform precision embedding scheme [17] enabling direct simulation of complex crack tip chemistry [97].

#### Mesoscopic and continuum models

The production of strain-induced vacancies has been modelled at the mesoscale level using dislocation dynamics as described in [33]. The authors incorporate vacancy nucleation and clustering into microvoids, which in turn induce cracking. Takai et al. [207] showed that the hydrogen adsorption capacity in ferrite is increased due to the hydrogen-enhanced production of strain-induced vacancies and dislocations. However, during annealing at 200 $^{\circ }$C, these defects were almost entirely annihilated, indicating that these were primarily vacancies. Models describing vacancy formation processes are also used to describe the loss in mechanical properties in the presence of hydrogen. For instance, Nagumo et al. [141] have described ductile fracture induced by vacancy clustering (leading to void formation) using a yield criterion originally developed for porous materials [63, 217]. They found that the values of the strain energy release rate (J-integral) in low-carbon steels are lower in the hydrogen-charged specimens, with an equivalent increase of 1.5% in the void volume fraction. Although they capture the degradation in the mechanical properties, it was not possible to directly link the effect of hydrogen with the kinetics of void formation and growth.

### Adsorption-induced decohesion mechanism AIDE

Based on the remarkable similarities between hydrogen-assisted cracking, stress corrosion cracking and adsorption-induced liquid–metal embrittlement observed for many materials, Lynch suggested that hydrogen-assisted cracking is also due to adsorption of hydrogen at the crack tip (adsorption-induced decohesion mechanism AIDE [121]. There is evidence of the embrittlement of metals (aluminium alloys, nickel, titanium alloys and magnesium) in aqueous or hydrogen environments, observed at crack velocities which are too high to allow the hydrogen to diffuse ahead of cracks. This phenomenon further supports a mechanism based on adsorption at crack tips. Metallographic and fractographic studies of crack growth show that environmentally assisted cracking occurs by a more localised plastic flow and microvoid coalescence process. Lynch concludes that environmentally assisted cracking occurs because adsorption facilitates the emission of dislocations from crack tips and thereby promotes the coalescence of cracks with voids ahead of the cracks. Evidence for a mechanism of embrittlement involving an adsorption-induced localised slip process is also observed in aluminium alloy 7075, high-strength steels and titanium [122].

The term "dislocation emission" in the AIDE model encompasses both nucleation and subsequent movement of dislocations away from the crack tip. The nucleation stage is critical and is facilitated by adsorption. It involves simultaneous formation of a dislocation core and surface step by breaking and reforming of interatomic bonds over several atomic distances. Thus, weakening of interatomic bonds by adsorbing hydrogen can facilitate the process. Once nucleated, dislocations can readily move away from the crack tip under the applied stress. Crack growth primarily occurs by dislocation emission from crack tips. The crack growth mechanisms in ductile materials can help us to understand why facilitating dislocation emission from crack tips results in embrittlement. In non-embrittling environments ductile crack growth occurs predominantly by egress of dislocations, nucleated in the plastic zone ahead of cracks, with little or no emission of dislocations occurring from crack tips. Dislocation emission from crack tips is difficult in such environments because interatomic bonding at crack tips is intrinsically strong. When dislocation egress around crack tips predominates, only a small proportion of dislocations emanating from near-crack-tip sources exactly intersect crack tips to produce crack advance. Most dislocations produce only blunting or contribute to the strain ahead of cracks. Large strains ahead of cracks are therefore needed to produce crack growth by microvoid coalescence. When hydrogen adsorption weakens interatomic bonds and thereby promotes dislocation emission from crack tips, a greater proportion of dislocation activity results in crack growth since dislocation emission produces crack advance as well as crack opening. Thus, coalescence of cracks with voids occurs at lower strains. However, to the authors’ knowledge there is no direct evidence of this phenomenon. In the AIDE model, crack growth occurs not only by dislocation emission from crack tips, but also involves nucleation and growth of microvoids ahead of crack tips. Nucleation and growth of voids at slip-band intersections, or other sites in the plastic zone ahead of cracks, occur because stresses required for dislocation emission are sufficiently high to induce some general dislocation activity ahead of cracks. Void formation contributes to crack growth, resharpening the crack tip and resulting in small crack-tip-opening angles. However, in the AIDE model dislocation emission is the primary crack growth mechanism. Hydrogen diffusion to, and adsorption at, internal crack tips or voids is necessary for AIDE. Crack paths produced as a result of the AIDE mechanism could be intergranular or transgranular depending on where dislocation emission and void formation occurs most easily. A combination of AIDE, HELP, and HID mechanisms could occur in a number of cases, depending on the material and other variables. For example, dislocations nucleated from crack tips (due to AIDE) may move away from crack tips more readily due to HELP, thereby decreasing the back-stress on subsequent dislocation emission. For crack growth predominantly by AIDE, void nucleation ahead of cracks could be promoted at slip-band intersections by HELP or by HID at particle-matrix interfaces. Increase in back-stresses from dislocations emitted due to AIDE can initiate HID, followed by AIDE again when the crack tip had moved away from the stress field of dislocations previously emitted. The AIDE mechanism in some materials is supported by the presence of high concentrations of hydrogen adsorbed on surfaces [169], surface-science observations [108], atomistic modelling [197] and metallographic and fractographic observations [122].

### Discussion and outlook on the HE mechanisms

Given the wide range of mechanisms reviewed above and the somewhat conflicting evidence in the literature, it is very difficult to give an opinion on how the various microscopic mechanisms (HID, HELP, HESIV, AIDE, etc.) combine to produce a macroscopic brittle fracture response. There is enough evidence although not proven experimentally, that in many situations a combination of two or more mechanisms is required to explain the embrittlement. In particular we have discussed the combination of HELP and HID in "Continuum models of the HID mechanism" section and of HELP and HESIV in "Atomistic simulations" section. Furthermore, we have presented in "Adsorption-induced decohesion mechanism AIDE" section a brief discussion on the possibility of the occurrence of a combination of HELP, HID and AIDE mechanisms.

The widely reported belief that high-strength steels are more prone to hydrogen embrittlement than simpler steels is contradicted by an elegant early work [162], demonstrating that embrittlement also affects low-strength pure iron. It is our belief that substantial progress can only be made by combining simulations and experimental work at different scales and, indeed, this is the philosophy underlying the HEmS research project on which the authors are presently working.

In common with the authors of other recent reviews [123, 178], we believe that the concerted action of a range of microscopic mechanisms provides the most likely explanation for hydrogen embrittlement. Nonetheless, we can identify a number of factors that might promote the occurrence of one mechanism over another: these include the local hydrogen concentration, dislocation density, phases present in the material and strain rate. The latter is particularly important, as many steels are strongly strain rate dependent.

The lack of standardisation of experimental procedure leads to problems when comparing results in the literature. We believe that designing a programme of key experiments is essential if we are to elucidate the contribution made by each of the microscopic mechanisms. For example, when analysing experimental results it is important to contrast the time taken for hydrogen to diffuse inside the specimen with the duration of the testing procedure itself. This can also impact on the design of experiments: the large variation in hydrogen diffusion rates when charging pure iron and high-strength steels necessitates varying the strain rate used in tensile tests.

## Hydrogen embrittlement mitigation strategies and design of new steels

It is understood that hydrogen that is trapped in steels does not contribute to the degradation of the material properties [18]. In fact early experiments have verified that diffusible hydrogen is responsible for the embrittlement process [86]. The ratio between immobilised and mobile hydrogen is highly dependant upon the steel’s microstructure, including key properties such as solubility, diffusivity and trapping behaviour. During the manufacturing process, there are several options for hydrogen control. In the molten state, a vacuum treatment can encourage degassing of otherwise solubilised gasses, including gaseous oxides, nitrogen and indeed hydrogen. This can be enhanced through the use of an inert gas-bubbling method, in order to further reduce the total hydrogen content in the melt [65]. Once cast, steels can then be annealed at sufficiently low temperatures to avoid side transformations whilst enhancing diffusion and thus out-gas hydrogen. However, whilst such an approach can limit the total hydrogen content, it does require long heat treatments at low temperatures, which are dependant on the diffusion path, and are thus specific to part geometries. This approach is designed to avoid so-called "intrinsic hydrogen", which, whilst important, does not control hydrogen that is introduced from other sources (sometimes referred to as "environmental hydrogen"). In general, it is important to control the quantity of diffusible hydrogen within the microstructure in order to ensure its resistance to embrittlement. This can be done by either: (a) preventing hydrogen ingress or (b) introducing an intrinsic resistance within the microstructure.

- Preventing or limitating hydrogen ingress in the material can be achieved by the application of a hydrogen-resistant coating, applied by methods such as electroplating or hot-dip galvanising processes. These treatments must be applied in an accurate manner; in fact the coating process itself can introduce hydrogen [109]. There are several viable methods in the literature, such as cadmium or Zn–Ni coating, or the deliberate formation of stable oxides, such as "Black oxide" magnetite (${\mathrm {Fe}}_3{\mathrm {O}}_4$) [79]. ${\mathrm {Fe}}_3{\mathrm {O}}_4$ in particular has been shown to reduce wear [172]; thus, this coating may be particularly useful in bearing applications. Other compounds, such as ${\mathrm {TiO}}_2$ [12], ${\mathrm {Al}}_2{\mathrm {O}}_3$ and ${\mathrm {Si_3N_4}}$ have been proposed [56, 211, 238]. Nitriding of the parent material’s surface layer has been successfully used in various austenitic stainless steels in order to limit hydrogen ingress [130, 137]. Furthermore, this process has been shown to decrease desorption and permeation of hydrogen in Armco irons [239]. This has also been demonstrated using TiN coatings on an austentic stainless steel substrate [181]. Cadmium applied by an electroplating method has a low penetration rate, owing to its lower diffusivity compared to ferrite. Furthermore, the electroplated surface layer can enhance the recombination rate of absorbed hydrogen, aiding egress rates [98]. However, due to the toxicity of cadmium, the use of cadmium coatings has a limited applicability. Moreover, it is unsuitable for e.g. marine applications. Lastly, the plating process electrolytically introduces hydrogen itself, and thus, a post-annealing step is required to remove the hydrogen, with typical times in the range of 8–24 h, at $200\,^{\circ }\hbox {C}$ [240]. Alternative electroplating materials include Zn and Zn–Ni have been proposed in the literature to provide a barrier for hydrogen ingress. However, there have been inconsistent findings related to their effectiveness [54], mainly due to coating defects. Regardless of the materials used for the coating in use, there is an underlying argument that the coating may not be suitable for the operating conditions required for the component. The coating must remain intact and adherent to the part in order to retain its hydrogen-resistant properties.
- Despite the application of a coating, hydrogen may still enter the microstructure and provide a route for embrittling processes. In this case, an intrinsic resistance may be considered when designing a material’s microstructure. It is necessary to provide sufficient trapping capabilities in order to absorb any hydrogen that may enter during the component’s in-service lifetime. Alternatively, degassing treatments used to release hydrogen during a part’s life cycle need to be considered [164, 166, 234]. If the traps saturate, then they may ultimately not mitigate hydrogen ingress. Hence, it is required that the material has a sufficient number density of traps to provide an appropriate uptake. As an example, bearings for off-shore wind turbines can accumulate up to 3 wppm hydrogen over their lifetime [177]. It is feasible to trap this amount of hydrogen through engineered microstructural traps [138] improving the hydrogen embrittlement resistance. Traps can take on a variety of forms, and this has been the subject of extensive research in the literature. A significant quantity of hydrogen can be trapped at dislocations; however, these traps are shallow and easily release hydrogen back into the matrix. Experimental work carried out by Takasawa et al. [209] and Hejazi et al. [71] suggested that a reduction in dislocation density and grain refinement is effective in reducing HE susceptibility. From these experiments, it is clear that "trapping" alone may be an insufficient predictor of HE experiments, as this is based upon a somewhat arbitrarily chosen binding energy cut-off [113]. However, it is important from a theoretical perspective to consider more complex local equilibrium scenarios [21], with both binding energy $E_{\mathrm{B}}$ and trap density $N_{{\mathrm{T}}_{\mathrm{B}}}$, which may better predict HE behaviour. A reduction of HE susceptibility through grain refinement was also suggested for drawn austenite [158]. It was reported that grain refinement minimised the formation of brittle phases. Grain boundary engineering has been proposed in FCC metals, with the aim of inducing a high density of low-$\Sigma$ grain boundaries, such as twin boundaries, through thermomechanical processing. Bechtle et al. [14] proposed that increasing the proportion of these boundaries in relation to high-$\Sigma$ boundaries can improve HE susceptibility in pure nickel. However, there are controversial results within the literature. Seita et al. [188] showed that, in Inconel 725 superalloys, coherent twin boundaries may have a dual role in hydrogen-assisted fracture. They used environmental SEM and in-situ tensile tests to show that $\Sigma 3$ coherent twin boundaries are preferential sites for crack initiation, whilst simultaneously impeding crack propagation. This dual role of low-$\Sigma$ grain boundaries was discussed to decrease grain boundary cohesive energy and promote decohesion. More recently, Takahashi et al. [205] investigated the local effects of gaseous hydrogen on grain boundary embrittlement in ${\hbox {Ni}}_3 {\hbox {Al}}$. They used a microsampling technique with nanoindentation inside an environmental TEM, observing different fracture phenomena between charged and H-free samples. At grain boundaries, the literature points to significantly different behaviours for any specific alloy system in presence of hydrogen. Due to the controversy in the effectiveness of grain boundary engineering to improve HE susceptibility, our understanding is that this mitigation method should be applied only in alloy systems where the effects of hydrogen on grain boundaries are indeed well understood. Similarly to grain boundary engineering, the manipulation of texture was proposed as a mitigation strategy against HE, relying on anisotropic effects. It was shown that texture has a significant effect on HE susceptibility in rolled pipeline steels exhibiting a mixed ferritic, bainitic and martensitic matrix [132, 222, 224]. Results show that in pipeline steels, texture with grain orientations in 111 plane parallel to the normal direction (ND) of steel rolling direction (${111} \parallel \hbox {ND}$) presented an increased resistance to hydrogen-induced cracking, whilst grains oriented with ${001} \parallel \hbox {ND}$ were prone to hydrogen cracking. Some textures (${110} \parallel \hbox {ND}$, ${332} \parallel \hbox {ND}$ and ${112} \parallel \hbox {ND}$), thought to be crack resistant textures, were involved in crack propagation. Evaluation of the effectiveness of microstructural engineering has been the subject of extensive discussions. For instance, Wang et al. [228] studied the mechanisms of hydrogen embrittlement in pure iron using repeated stress-relaxation tests under electrochemical charging. A significant increase in dislocation activity with increasing H content was observed before undergoing intergranular failure, wherein decohesion was considered; however, HELP was considered to be the primary mechanism. Similar support for this comes from Neeraj et al. [147] in a dual ferrite/pearlite microstructure, in conjunction with vacancy generation (HESIV). Nagao et al. [139] considered both HID and HELP in lath-martensitic steels. These seemingly disparate observations show the difficulty of engineering a solution to limit HE using "ground-up" approaches. It is thus clear that several factors need to be considered in detail when evaluating the embrittlement resistance of materials and components. Although coatings can be applied to almost any material, their use is limited by, for example, wear and coating lifetime. Similarly, intrinsic protection through microstructural engineering must similarly not negatively impact a material’s mechanical properties.

We believe it can be said that there are a number of both positive and contrapositive indications for particular microstructures with respect to HE performance. As in the prior example of Inconel, texture orientation was tested; however, this was only tested in the context of pipeline steels. What is clear, however, is that any attempt to engineer microstructural traps will only be of benefit if there are of sufficiently high number density, and that this must not come at the cost of mechanical properties. In the absence of any direct engineering predictive capability from a theoretical perspective, specific engineering attempts must be made with knowledge of the intended application, the expected in-service hydrogen loading and the material’s mechanical performances in the presence of hydrogen.

## Open questions and outlook

In this paper we have presented a review up to the current state of the art on the effect of hydrogen in the degradation of steels. The main focus is to give the reader a comprehensive understanding of hydrogen embrittlement through an overview of experimental and modelling studies as well as theoretical evidence from the atomistic to the continuum scale. Furthermore, we have dedicated a section to current mitigation strategies used to prevent hydrogen ingress and to design steel microstructures resistant to hydrogen embrittlement.

Whilst there has been considerable progress in recent years, stimulated both by experimental insight and by modelling at the atomic and continuum scales, there are a number of key open questions that remain. One of the main issues is that there is still considerable disagreement in the scientific literature concerning the underlying processes that are responsible for hydrogen embrittlement, even in simple material systems. In our opinion, a comprehensive understanding of hydrogen embrittlement is essential to underpin design strategies for the next generation of ultra-high-strength steels.

In the authors’ opinion further research effort should be devoted to a more in-depth understanding of how hydrogen affects mechanical performance by integrating a wide range of computational modelling schemes and experimental techniques at different scales. The new understanding derived from these studies might guide the development of new procedures for the design of steel microstructures that are resistant to HE.

In particular, a significant research effort is underway by the authors into computational modelling schemes that exploit machine learning, atomistic simulation, kinetic Monte Carlo, irreversible thermodynamics, statistical physics, theoretical chemistry and finite element simulation, to study several paradigms of hydrogen diffusion, trapping and embrittlement.

From an experimental point of view, significant contributions have been made in the field of imaging techniques for deuterium at the atomic scale. We have developed a new advanced APT technique for imaging of deuterium in steels which will give important insight in the characterisation of traps. We are currently working on advanced ultra-high-strength steels that are being developed at the SKF UTC in Cambridge.

Our main goal is to build an extensive database of atomic, defect, thermodynamic, mesoscopic and continuum properties relevant to the embrittlement process, and identifying the full range of traps for hydrogen in microstructures. Together, these developments would enable engineering models for diffusion, plasticity, interfaces and fracture based on a series of atomistic, thermostatistic and mesoscopic models.

These models will of course need comprehensive validation over a wide range of nano-, meso- and macroscale characterisation and testing procedures such as atom probe, which holds great promise, e.g. to identify the arrangement of hydrogen within traps. Such improved understanding could be extremely important in order to identify microstructures that are resistant to hydrogen embrittlement and thus to develop new procedures for alloy design that take into account the influence of H on mechanical performance, leading to ultra-high-strength steels that are resistant to hydrogen embrittlement.

## List of symbols

TDA: Thermal desorption analysis
APT: Atom probe tomography
$K_{t}$: Stress intensity factor
IG: Intergranular fracture
HID: Hydrogen-induced decohesion
HELP: Hydrogen-enhanced local plasticity
HIPT: Hydrogen-induced phase transformation
HESIV: Hydrogen-enhanced strain-induced vacancy formation
AIDE: Adsorption-induced dislocation emission
SANS: Small-angle neutron scattering
TEM: Transmission electron microscopy
CZM: Cohesive zone modelling
CMD: Centroid molecular dynamics
SFE: Stacking fault energy
PIF: Path-integral formulation
RPMD: Ring-polymer molecular dynamics
DFT: Density functional theory
kMC: Kinetic Monte Carlo
MC: Monte Carlo
EAM: Embedded atom model
SSRT: Slow strain rate test
TRIP: Transformation-induced plasticity
$\theta _{\mathrm{l}}$: Hydrogen occupancy in the lattice
$\theta _{\mathrm{t}}$: Hydrogen occupancy in trapping sites
$$N_{\mathrm{t}}$$: Trap density
$$N_{\mathrm{l}}$$: Lattice density
$C_{\mathrm{l}}$: Hydrogen concentration in the lattice
$C_{l_{0}}$: Reference hydrogen concentration
$C_{\mathrm{t}}$: Hydrogen concentration in trapping sites
DS: Devanathan Stachurski
$$D_{\mathrm{L}}$$: Lattice diffusivity
$$D_{\mathrm{eff}}$$: Effective diffusivity
$t_{\mathrm{lattice}}$: Lag time in an ideal lattice
$t_{\mathrm{trap}}$: Lag time in traps
SKP: Scanning Kelvin probe
SKPFM: Scanning Kelvin probe force microscopy
BCC: Body-centred cubic
FCC: Face-centred cubic
FCT: Tetragonally distorted FCC
BCT: Body-centred tetragonal
HCP: Hexagonal close packed
$$\alpha $$: BCC phase
$$\gamma $$: FCC phase
$\gamma _{\mathrm{Hydride}}$: FCC hydride phase
$$\epsilon $$: HCP phase
$\epsilon _{\mathrm{martensite}}$: FCC $\gamma$ transformed in HCP phase
$$\epsilon _{\mathrm{p}}$$: Plastic strain
$K_{\mathrm{min}}$: Stress intensity factor at minimum loading
$K_{\mathrm{max}}$: Stress intensity factor at maximum loading
$$R_{K}$$: $$\frac{K_{\mathrm{min}}}{K_{\mathrm{max}}}$$
$$\Delta _{K}$$: $$K_{\mathrm{max}}-K_{\mathrm{min}}$$
$n_{0}$: Total number of chemical bonds
$n_{\mathrm{H}}$: Distribution of hydrogen by chemical bonds
$K'$: Stress intensity factor increment
$$\sigma _{Y}$$: Flow stress
$\sigma _{0}^{\mathrm{H}}$: Yield strength in the presence of hydrogen
$$\sigma _{\mathrm{h}}$$: Hydrostatic stress
$$\sigma _{\mathrm{e}}$$: Effective stress

## Footnotes

1 H is in principle diffusible so we also refer to H atoms in the lattice as "diffusing" hydrogen.

2 Hydrogen-induced decohesion is sometimes referred to as hydrogen-enhanced decohesion (HEDE).
